# A Universal Metal–Flavonoid Coating Strategy: Engineering Biomaterials for Diabetic Bone Regeneration

**DOI:** 10.1002/advs.202522509

**Published:** 2026-01-08

**Authors:** Chen Yang, Chenle Dong, Lefeng Su, Zhiqiang Liu, Lingyi Hu, Qishu Jin, Hao Chen, Chunlong Zhang, Yihao Wu, Jiang Chang, Zhaowenbin Zhang, Jiandong Yuan

**Affiliations:** ^1^ Department of Orthopaedics, Joint Centre of Translational Medicine The First Affiliated Hospital of Wenzhou Medical University Wenzhou Zhejiang China; ^2^ Zhejiang Engineering Research Center For Tissue Repair Materials Wenzhou Institute University of Chinese Academy of Sciences Wenzhou Zhejiang China; ^3^ Orthopedic Institute The First Affiliated Hospital Suzhou Medical College Soochow University Suzhou Jiangsu China; ^4^ Institute of Reproductive Health Tongji Medical College Huazhong University of Science and Technology Wuhan Hubei China; ^5^ Medical College Yangzhou University Yangzhou Jiangsu China; ^6^ State Key Laboratory of Advanced Fiber Materials College of Biological Science and Medical Engineering Donghua University Shanghai China

**Keywords:** copper homeostasis, diabetic bone, metal–flavonoid coating, quercetin

## Abstract

Under metabolic disorders such as diabetes, the regenerative capacity of bone tissue is compromised, characterized by hyperglycemia‐induced oxidative stress, impaired osteogenesis, and dysregulated angiogenesis. These complications undermine the efficacy of conventional bone repair materials, limiting their capacity to promote effective healing. Herein, we developed a metal–flavonoid functionalized coating strategy that integrates osteogenic and angiogenic metal ions with natural antioxidant flavonoids. Leveraging their chelation ability and strong surface affinity, a one‐pot approach is established to endow conventional bone repair materials with tailored biofunctions that modulate the diabetic bone microenvironment and facilitate regeneration. Among the tested candidates, copper‐quercetin (CQ) coating is screened as the optimal formulation owing to its potent antioxidative, osteoinductive, and pro‐angiogenic properties under high‐glucose (HG) conditions. Conventional bone repair materials (e.g., β‐tricalcium phosphate, β‐TCP) modified with this coating significantly ameliorated oxidative stress, restored osteogenesis, and rescued angiogenesis impaired by persistent hyperglycemia. In diabetic rats, CQ‐coated β‐TCP (β‐TCP@CQ) accelerated bone healing by 1.68‐fold compared to unmodified controls. Mechanistically, the CQ coating activated the ATP7A/SOD3/FLT1 axis to restore copper homeostasis in bone marrow mesenchymal stem cells while stimulating the PI3K‐Akt pathway to enhance osteogenic differentiation. Moreover, the coating exhibited broad versatility across multiple biomaterial compositions (metals, ceramics, polymers, and composites) and structures (2D discs, 3D scaffolds). This metal–flavonoid coating strategy demonstrated feasibility and scalability, while also providing a mechanistic foundation and technical platform for the effective use of conventional bone repair materials in complex pathological contexts.

## Graphical abstract

Construction of metal–flavonoid functionalized coatings on conventional bone repair materials. A one‐pot method is used to directly construct multifunctional metal–flavonoid coatings on the surface of traditional bone repair materials by selecting metal ions with osteogenic/angiogenic properties and flavonoids with reactive oxygen species scavenging capabilities. This approach enhances the bone regeneration and repair capacity of conventional bone repair materials in diabetic pathological conditions.

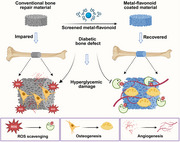

## 1 Introduction

Diabetes represents one of the most pressing global health challenges of the 21st century. According to the most recent statistics from the International Diabetes Federation, approximately 589 million adults aged 20–79 were affected worldwide in 2024, accounting for 11.1% of the population in this age group [1]. As a chronic metabolic disorder, diabetes leads to multiorgan dysfunction, and within the skeletal system it markedly increases fracture risk [2]. In particular, individuals with type II diabetes exhibit a 1.2–1.7‐fold higher incidence of hip and spinal fractures compared with nondiabetic individuals [3, 4]. Beyond elevating fracture risk, diabetes frequently results in delayed healing or nonunion of bone defects, thereby severely impairing patient quality of life and clinical outcomes [5]. At present, no bone‐regenerative materials have been specifically developed for diabetic patients in clinic. Considering the urgent clinical need and the complexity of fabricating new implantable biomaterials, surface functionalization of existing implants with bioactive coatings has emerged as a practical and efficient strategy [6, 7]. Such modifications can confer novel biological functions while preserving intrinsic mechanical properties, thus improving bone regeneration under diabetic pathological conditions.

A thorough understanding of the pathological microenvironment in diabetic bone defects is essential for designing targeted functional coatings. Long‐term hyperglycemia induces multiple deleterious alterations that collectively impair bone regeneration, creating a major barrier to healing. Chronic hyperglycemia drives excessive accumulation of reactive oxygen species (ROS), persistent inflammation, and increased production of advanced glycation end products (AGEs) [8]. These factors mutually reinforce one another, forming a vicious cycle that disrupts the function of stem cells, osteoblasts, and osteoclasts, ultimately impairing bone repair. In addition, angiogenesis is compromised under diabetic conditions, resulting in impaired microcirculation, insufficient oxygen and nutrient supply, and disruption of osteo‐angiogenic coupling, all of which exacerbate the challenge of bone regeneration [8]. Therefore, the central objective of functional coatings should be to restore osteogenic and angiogenic potential within the diabetic microenvironment, which is critical for effective repair.

Flavonoids (e.g., luteolin, curcumin, and quercetin) are natural polyphenolic compounds that have attracted considerable attention owing to their diverse biological activities [9]. Owing to their abundant phenolic hydroxyl groups and conjugated structures, flavonoids exhibit potent free radical‐scavenging capabilities. Acting as exogenous antioxidants, they eliminate excessive ROS, reduce oxidative stress, and maintain a reductive intracellular environment that supports osteogenic differentiation [10]. In this way, flavonoids mitigate the inhibitory effects of oxidative stress on both osteogenesis and angiogenesis. Moreover, flavonoids function as natural metal chelators. Their rich coordination groups and environmental responsiveness enable high‐affinity binding with multiple biologically active ions [11]. For instance, the 3′,4′‐ortho‐dihydroxy structure in luteolin and quercetin, and the β‐diketone/enol group in curcumin confer strong metal–chelating capacity [12]. Numerous studies have confirmed that metal ions such as magnesium ion (Mg^2+^), cobalt ion (Co^2+^), copper ion (Cu^2+^), zinc ion (Zn^2+^), and strontium ion (Sr^2+^) play pivotal roles in bone regeneration [13]. These bioactive ions promote osteogenesis and angiogenesis through diverse signaling pathways when administered at appropriate concentrations. For example, Sr^2+^ enhances osteoblast‐mediated bone matrix synthesis while inhibiting osteoclast‐mediated bone resorption, thereby promoting net bone formation, whereas Cu^2+^ supports collagen crosslinking, antioxidant defense, and endothelial cell mediated vascularization [14, 15].

Importantly, flavonoids display strong interfacial affinity and can adsorb onto a wide range of substrates, including metals, ceramics, and polymers, through hydrogen bonding, π–π stacking, or hydrophobic interactions [16, 17]. Upon coordination with metal ions, interfacial complexes are rapidly formed, enabling the construction of 3D metal–flavonoid network structures via layer‐by‐layer self‐assembly [18, 19]. These nanocoatings exhibit dynamic dissociation in response to environmental changes and frequently display greater stability and bioactivity than individual flavonoid components [18]. By integrating the complementary functions of metal ions and flavonoids with the controllability of their assembly, recent studies have suggested that metal–flavonoid or metal–polyphenol complexes can promote the repair of pathological bone defects, including those associated with diabetes and osteoporosis [20, 21]. Collectively, these findings highlight that rationally engineered metal–flavonoid coatings may serve as multifunctional biointerfaces that integrate antioxidation, osteogenesis, and angiogenesis, thereby offering a transformative strategy to enhance bone regeneration under diabetic conditions.

**SCHEME 1:**
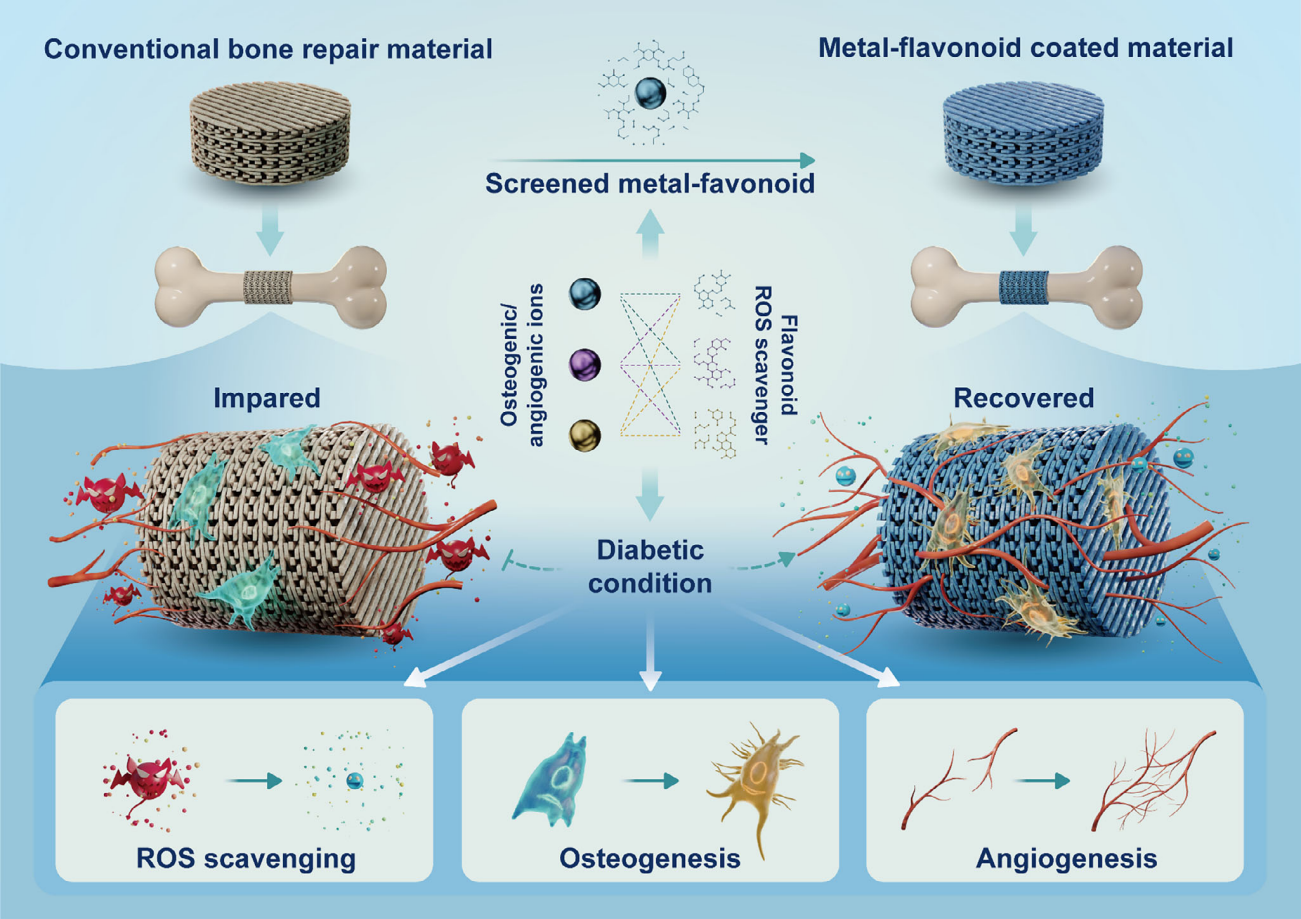
Construction of metal–flavonoid functionalized coatings on conventional bone repair materials and their application in diabetic bone regeneration. A one‐pot method was used to directly construct multifunctional metal–flavonoid coatings on the surface of traditional bone repair materials by selecting metal ions with osteogenic/angiogenic properties and flavonoids with ROS scavenging capabilities. This approach enhances the bone regeneration and repair capacity of conventional bone repair materials in diabetic pathological conditions.

Based on this rationale, the present study hypothesizes that specific metal ions combined with suitable flavonoid molecules can form multifunctional surface coatings that significantly improve the performance of conventional bone repair materials, such as β‐tricalcium phosphate (β‐TCP), in diabetic bone defects. To test this hypothesis, bioactive metal ions (Mg^2+^, Co^2+^, Cu^2+^, Zn^2+^, and Sr^2+^), recognized for their osteogenic and angiogenic effects [22], together with flavonoid compounds (luteolin, curcumin, and quercetin) known for potent reactive oxygen species (ROS)‐scavenging activity [23], were used to generate diverse metal–flavonoid combinations. These combinations were systematically screened to identify those with optimal synergy under hyperglycemic conditions. The assembly mechanisms, biological activities, and molecular pathways were comprehensively investigated, and both in vitro and in vivo models were employed to validate therapeutic efficacy (Scheme 1). This work introduces a promising surface functionalization strategy that unifies antioxidation, osteogenesis, and angiogenesis within a single bioactive interface. By integrating the intrinsic bioactivities of flavonoids with the essential signaling roles of metal ions, the study establishes a generalizable coating platform with broad translational potential, offering a new path toward treating diabetic bone defects and other chronic metabolic disorders.

## 2 Results

### 2.1 Construction, Screening, and Formation Mechanism of Metal–Flavonoid Coatings

**FIGURE 1:**
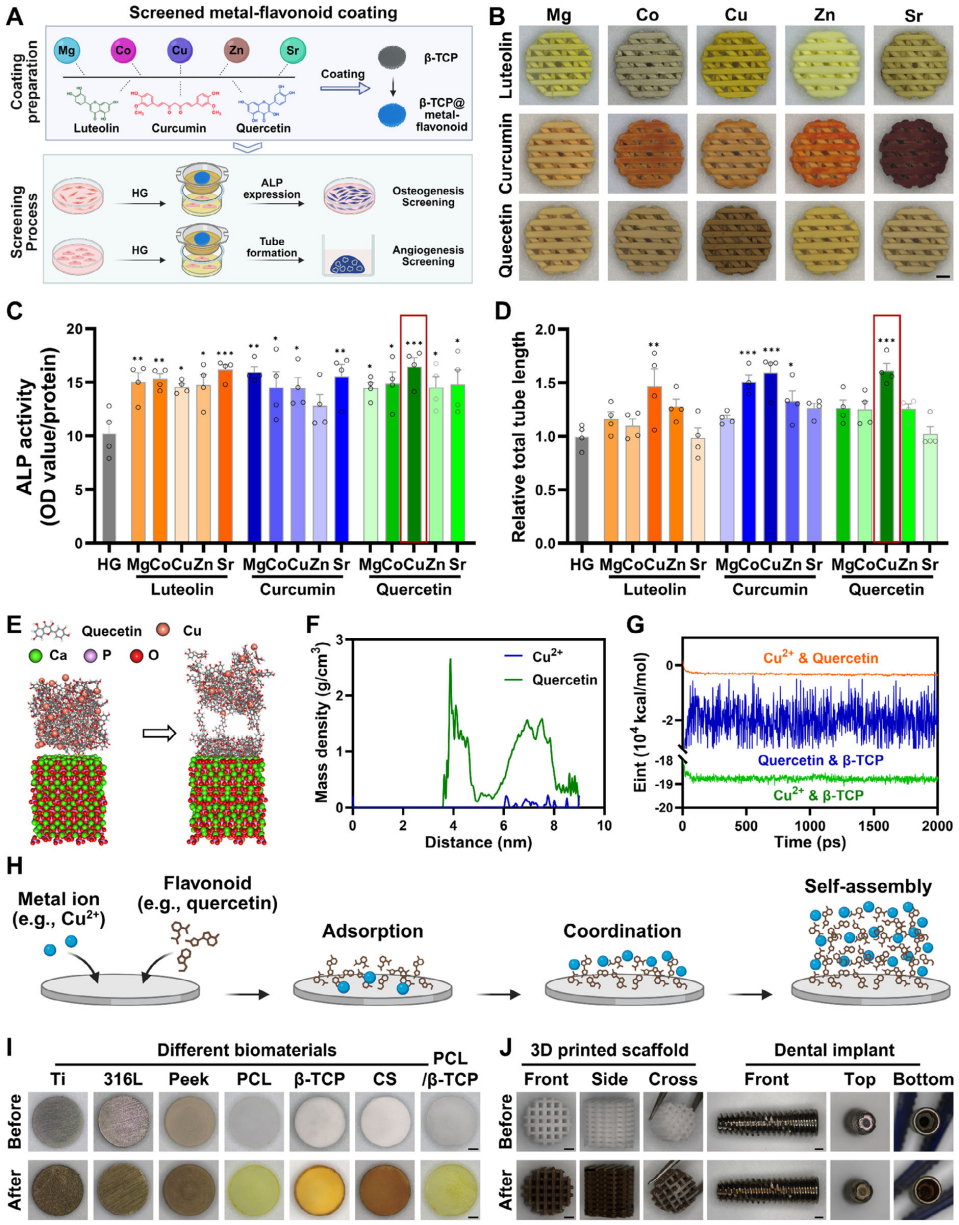
Construction, screening, and formation mechanism of metal–flavonoid coatings. (A) Schematic diagram of the screening process for metal–flavonoid coatings. (B) Macroscopic images of β‐TCP modified with self‐assembled coatings composed of various metal ions and flavonoids. Scale bar: 1 mm. (C) ALP activity of BMSCs cultured with different metal–flavonoid coated β‐TCP scaffolds under HG conditions. *n* = 4. (D) Total tube length formed by HUVECs cultured with different metal–flavonoid coated β‐TCP scaffolds under HG conditions. *n* = 4. (E) Molecular dynamics simulation of CQ formation on the β‐TCP surface. (F) Relationship between Cu^2+^ and quercetin density in the system and their respective distances from the β‐TCP surface. (G) Temporal variation in the interaction energy between Cu^2+^, quercetin, and β‐TCP. (H) Schematic representation of the CQ coating formation process. (I,J) Universal verification of CQ coating on different types and architectures of biomaterials. Scale bar: 1 mm. * indicates significant difference compared to the HG group. *p < 0.05, **p < 0.01, ***p < 0.001.

To construct metal–flavonoid coatings on the surface of conventional bone repair materials for the treatment of diabetic bone defects, β‐tricalcium phosphate (β‐TCP) was selected as a representative material. Mg^2+^, Co^2+^, Cu^2+^, Zn^2+^, and Sr^2+^ were selected as representative osteogenic/angiogenic metal ions, whereas luteolin, curcumin, and quercetin were chosen as representative flavonoids. The osteogenic activity of β‐TCP with different coating modifications was evaluated in bone marrow mesenchymal stem cells (BMSCs), and the angiogenic activity was assessed in human umbilical vein endothelial cells (HUVECs) under high‐glucose (HG) conditions to identify the optimal coating formulation (Figure 1A).

Before constructing the various metal–flavonoid coatings, the preparation conditions were optimized using copper‐quercetin (CQ) on β‐TCP discs as a model system. Quercetin was found to fully dissolve in alkaline aqueous solution at pH ≥ 10 (Figure S1). On this basis, the ratio of Cu ions to quercetin was optimized, and a Cu‐to‐quercetin molar ratio of 1:2 yielded the most prominent CQ coating on the β‐TCP surface (Figure S2). Using this optimal ratio, the effects of CQ concentration, coating duration, and coating cycles on β‐TCP modification were further investigated. Macroscopic images revealed that the color intensity of the CQ coating increased with higher CQ concentration, longer coating duration, and additional coating cycles (Figures S3–S5), confirming the feasibility of this coating strategy.

Subsequently, various metal–flavonoid combinations were applied to β‐TCP scaffolds to screen for osteogenic and angiogenic potential. Although the colors of the different metal–flavonoid coatings varied, all scaffolds exhibited uniform surface coverage (Figure 1B). BMSCs were then co‐cultured with the various coated scaffolds under HG conditions. All scaffolds significantly enhanced the Alkaline phosphatase (ALP) activity of BMSCs, with Sr‐luteolin and CQ demonstrating the strongest effects, increasing ALP activity by 57.8% and 60.6%, respectively (Figure 1C). In terms of in vitro angiogenic potential, Cu‐curcumin and CQ exhibited the best performance, increasing total tube length by 59.3% and 61.3%, respectively, compared with the HG group (Figure 1D). In summary, the CQ coating showed the most pronounced osteogenic and angiogenic potential under HG conditions.

To further investigate the binding mechanism between the CQ coating and β‐TCP, molecular dynamics (MD) simulations were performed by constructing a system comprising Cu, quercetin, and a β‐TCP crystal with an exposed [001] plane. Conformational analysis revealed that Cu^2+^ first adsorbed onto the β‐TCP surface and then coordinated with the phenolic hydroxyl groups of quercetin, enabling molecular growth along the crystal plane (Figure 1E and Figure S6). Moreover, conjugation between the benzene ring of quercetin and the β‐TCP surface promoted continuous stacking, further facilitating CQ growth on the crystal surface and forming a coating approximately 4 nm thick (Figure 1F). To elucidate the interactions among the molecules in the system, the binding energies of Cu, quercetin, and the β‐TCP crystal surface were calculated (Figure 1G). All binding energy values were negative, indicating that adsorption of Cu^2+^ or quercetin onto the β‐TCP surface, as well as self‐assembly of Cu^2+^ and quercetin, occurred spontaneously. Furthermore, the binding energy of Cu^2+^ or quercetin to the β‐TCP surface was substantially greater than that between Cu^2+^ and quercetin themselves, suggesting that Cu^2+^ and quercetin preferentially adsorb onto the β‐TCP surface, coordinate with each other, and subsequently self‐assemble into the CQ nanoscale coating (Figure 1H).

Finally, to demonstrate the generality of this formation mechanism, the coating capability of CQ was validated on bone repair materials with diverse chemical compositions and architectures (Figure 1I,J). The results demonstrated that a uniform CQ coating formed on the surfaces of diverse biomaterials, including metals such as titanium (Ti) and 316L stainless steel; polymers such as polyether ether ketone (PEEK) and polycaprolactone (PCL); ceramics such as β‐TCP and calcium silicate (CS); and composites, such as PCL/β‐TCP. Furthermore, uniform CQ coatings were also formed on complex structural materials, such as 3D porous scaffolds and clinical implants, fully demonstrating the versatility of this strategy.

### 2.2 Characterization of the Screened CQ Coated β‐TCP (β‐TCP@CQ) Scaffold

**FIGURE 2:**
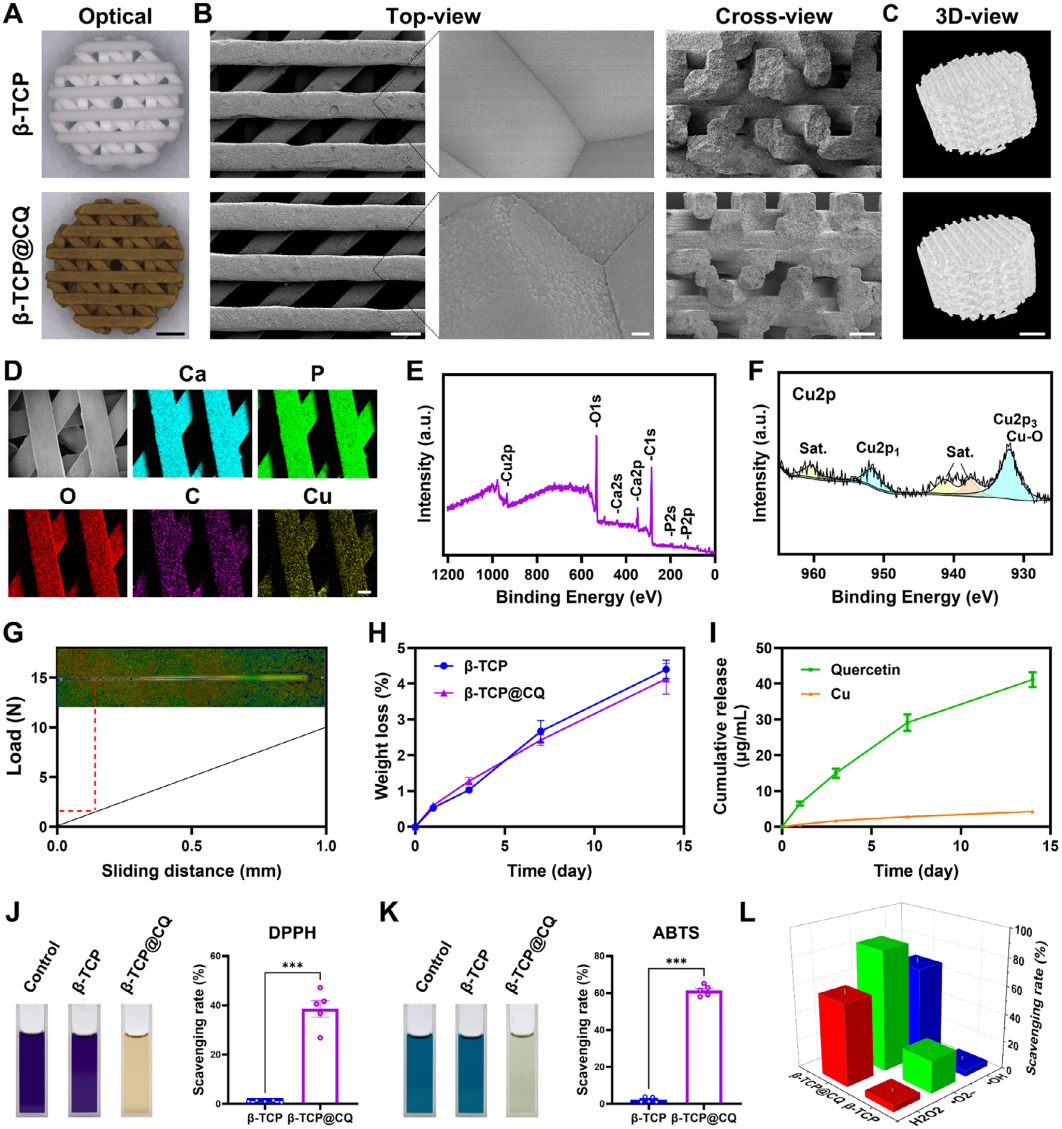
Characterization of the screened CQ coated β‐TCP (β‐TCP@CQ) scaffold. (A) Representative macroscopic photograph of β‐TCP and β‐TCP@CQ. Scale bar: 1 mm. (B) Representative SEM images of β‐TCP and β‐TCP@CQ. Scale bar: 0.5 mm and 200 nm for Top‐view; 0.5 mm for Cross‐view. (C) Representative micro‐CT image of β‐TCP and β‐TCP@CQ. Scale bar: 0.5 mm. (D) Elemental distribution of β‐TCP@CQ assessed using EDS, confirming the presence of Ca, P, O, C, and Cu elements. Scale bar: 0.2 mm. (E) XPS survey spectra of β‐TCP@CQ. (F) High resolution XPS spectrum of Cu2p in β‐TCP@CQ. (G) Coating bonding strength evaluation of β‐TCP@CQ via nano‐scratch testing. (H) Assessment of the degradation performance of β‐TCP and β‐TCP@CQ. *n* = 4. (I) Quercetin and Cu^2+^ ion release profiles of β‐TCP@CQ over time. *n* = 4. (J,K) Radical scavenging activity of β‐TCP and β‐TCP@CQ against DPPH and ABTS•^+^ radicals. *n* = 5. (L) Scavenging ability of β‐TCP and β‐TCP@CQ against ROS, including H_2_O_2_, •O_2_^−^, and •OH. *n* = 5.

Subsequently, the physicochemical properties and antioxidant capacity of the screened β‐TCP@CQ scaffold were comprehensively evaluated. Macroscopic images revealed that, compared with pristine β‐TCP, a uniform brownish yellow CQ coating formed on the surface of the β‐TCP@CQ scaffold (Figure 2A). Scanning electron microscopy (SEM) further showed that the surface of β‐TCP was relatively smooth and flat, whereas the β‐TCP@CQ scaffold displayed a rough and uniform CQ coating (Figure 2B). Microcomputed tomography (micro‐CT) analysis demonstrated that the CQ coating did not alter the internal pore architecture of β‐TCP, preserving its porosity (Figure 2C and Figure S7). X‐ray diffraction (XRD) confirmed the high purity of β‐TCP, with diffraction peaks matching standard PDF#09‐0169 (Figure S8). Although no distinct differences were detected between the XRD patterns of β‐TCP and β‐TCP@CQ, energy‐dispersive X‐ray spectroscopy (EDS) revealed the presence of calcium (Ca), phosphorus (P), oxygen (O), carbon (C), and copper (Cu) in the β‐TCP@CQ scaffold. The detection of C and Cu further confirmed successful incorporation of the CQ coating (Figure 2D). Additionally, X‐ray photoelectron spectroscopy (XPS) analysis confirmed the presence of Ca, P, O, C, and Cu, with Cu existing in the Cu^2+^ state, indicating its coordination with quercetin through Cu^2+^ (Figure 2E,F and Figure S9).

To verify chelation between Cu^2^⁺ and quercetin, Fourier transform infrared spectroscopy (FTIR) and ultraviolet visible (UV–Vis) absorption spectroscopy were employed. FTIR analysis revealed shifts or enhancements of peaks at 1630, 1520, and 1270 cm^−1^, suggesting alterations in the carbonyl, phenyl, and hydroxyl groups of quercetin upon Cu coordination. A new peak at 606 cm^−1^ confirmed the presence of Cu*─*O stretching vibrations, further supporting the formation of a Cu^2+^‐quercetin chelate (Figure S10). UV–vis spectra revealed that quercetin exhibited strong absorption peaks at 270, 324, and 382 nm, which shifted to 262, 332, and 406 nm upon binding with Cu ions, confirming the formation of the Cu^2+^‐quercetin chelate in the CQ coating (Figure S11).

Furthermore, the compressive strength and Young's modulus of β‐TCP@CQ was evaluated and found to be slightly higher than that of pure β‐TCP, although the difference was not statistically significant (Figure S12). The interfacial bonding strength between the CQ coating and β‐TCP was assessed using a nanoscratch test. The results indicated that the bonding force between the CQ coating and the β‐TCP substrate was approximately 2.2 N, demonstrating strong adhesion (Figure 2G). More importantly, the β‐TCP@CQ scaffold exhibited stable degradation, with a biodegradation rate of approximately 4.13 wt% over 14 days (Figure 2H). During degradation, the color of the β‐TCP@CQ scaffold slightly faded, but no coating delamination was observed, confirming coating stability (Figure S13). Moreover, the β‐TCP@CQ scaffold exhibited sustained release of Cu^2+^ ions and quercetin over time. After 14 days, the cumulative release of quercetin reached 41.15 ± 4.22 µg/mL, whereas that of Cu^2+^ ions reached 4.26 ± 0.79 µg/mL (Figure 2I).

Notably, the β‐TCP scaffold, lacking intrinsic free radical scavenging ability, was compared with the β‐TCP@CQ scaffold, which exhibited significant scavenging activity against 1,1‐diphenyl‐2‐picrylhydrazyl (DPPH) and 2,2′‐azinobis(3‐ethylbenzothiazoline‐6‐sulfonic acid) (ABTS) radicals, with scavenging rates of approximately 38% and 61%, respectively (Figure 2J,K). In addition, the β‐TCP@CQ scaffold exhibited significant scavenging ability against multiple types of ROS, including hydrogen peroxide (H_2_O_2_), superoxide anion radicals (•O_2_^−^), and hydroxyl radicals (•OH), with scavenging rates of approximately 58%, 86%, and 65%, respectively (Figure 2L).

### 2.3 CQ Coating Enhanced the Angiogenic Potential of β‐TCP Under HG Conditions

**FIGURE 3:**
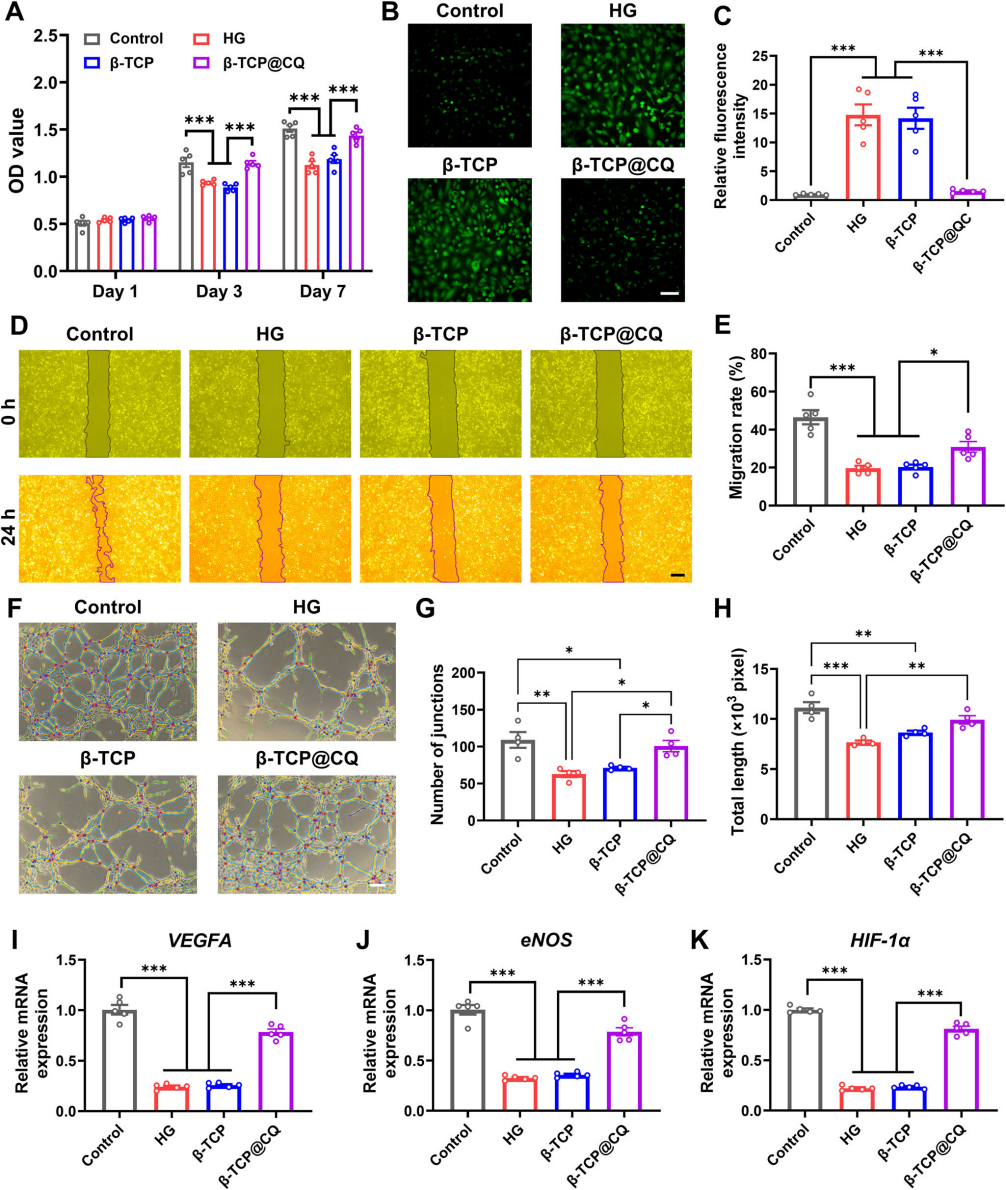
CQ coating enhanced the angiogenic potential of β‐TCP under HG conditions. (A) Viability of HG‐injured HUVECs treated with β‐TCP or β‐TCP@CQ scaffolds for 1, 3, and 7 days. *n* = 5. (B,C) Intracellular ROS levels in HG‐injured HUVECs treated with β‐TCP or β‐TCP@CQ scaffolds, detected by DCFH‐DA staining and corresponding quantitative analysis. *n* = 5. Scale bar: 100 µm. (D,E) Migratory capacity of HG‐injured HUVECs treated with β‐TCP or β‐TCP@CQ scaffolds. The green and yellow regions are pseudocolors added for visualization purposes. Scale bar: 100 µm. (F–H) Representative tube formation images and corresponding quantitative analysis of HG‐injured HUVECs treated with β‐TCP or β‐TCP@CQ scaffolds. *n* = 4. Scale bar: 100 µm. (I–K) qRT‐PCR analysis of the expression of typical pro‐angiogenic factors (*VEGFA*, *eNOS*, *HIF‐1α*) in HG injured HUVECs treated with β‐TCP or β‐TCP@CQ scaffolds. *n* = 5.

In the treatment of diabetic bone defects, promoting endothelial cell activation under HG conditions is essential for establishing a functional vascular network and ensuring adequate nutrient supply to the injured tissue. To this end, the pro‐angiogenic effects of β‐TCP@CQ on HUVECs under HG conditions were evaluated. Compared with the Control group, HUVEC viability was significantly reduced in the HG group on both Day 3 and Day 7. However, treatment with β‐TCP@CQ markedly reversed this decline, demonstrating a strong protective effect on cell viability. In contrast, β‐TCP alone did not exert a comparable effect, and HUVEC viability in the β‐TCP group remained similar to that in the HG group (Figure 3A). Given that the HG environment induces excessive ROS production in HUVECs, a major cause of reduced cell viability, intracellular ROS levels were assessed using DCFH‐DA staining (Figure 3B). β‐TCP alone was ineffective in scavenging ROS, with no significant difference in ROS levels observed between the β‐TCP group and the HG group. In contrast, β‐TCP@CQ significantly reduced ROS accumulation in HUVECs under HG conditions. Quantitative analysis showed that ROS levels in β‐TCP@CQ‐treated HUVECs were approximately 9‐fold lower than those in both the HG and β‐TCP groups, with no significant difference compared with the Control group (Figure 3C).

In addition, the HG environment impaired HUVEC migratory ability. However, β‐TCP@CQ significantly enhanced HUVEC migration, resulting in markedly higher migration rates compared with both the HG and β‐TCP groups (Figure 3D,E). Furthermore, although the HG environment inhibited tube formation in HUVECs, β‐TCP@CQ promoted robust angiogenesis under HG conditions (Figure 3F). Quantitative analysis demonstrated that β‐TCP@CQ markedly increased both the number of vascular junctions and the total vascular length, further confirming its pro‐angiogenic effect (Figure 3G,H). Moreover, quantitative real‐time polymerase chain reaction (qRT‐PCR) analysis revealed that β‐TCP@CQ significantly restored the expression of key angiogenic genes, including *VEGFA*, *eNOS*, and *HIF‐1α*, which were suppressed under HG conditions. In contrast, no significant differences in the expression of these genes were observed between the HG and β‐TCP groups (Figure 3I–K). Collectively, these results indicate that the CQ coating markedly alleviated HG‐induced angiogenic inhibition and enhanced the pro‐angiogenic potential of β‐TCP under hyperglycemic conditions.

### 2.4 CQ Coating Enhanced the Osteogenic Potential of β‐TCP Under HG Conditions

**FIGURE 4:**
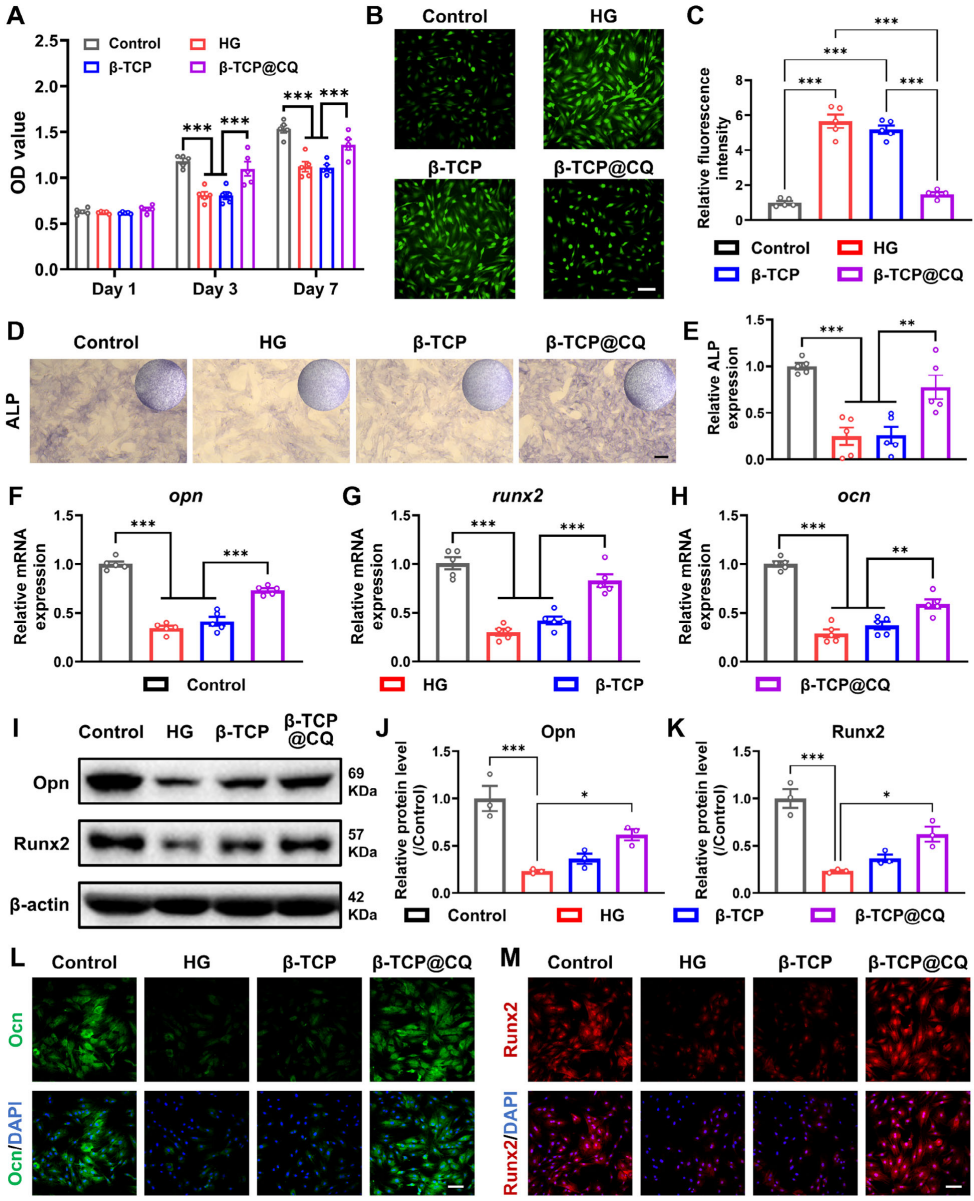
CQ coating enhanced the osteogenic potential of β‐TCP under HG conditions. (A) Viability of HG‐injured BMSCs treated with β‐TCP or β‐TCP@CQ scaffolds for 1, 3, and 7 days. *n* = 5. (B,C) Intracellular ROS levels in HG‐injured BMSCs treated with β‐TCP or β‐TCP@CQ scaffolds, detected by DCFH‐DA staining and corresponding quantitative analysis. *n* = 5. Scale bar: 100 µm. (D,E) ALP staining and corresponding semiquantitative analysis of HG‐injured BMSCs treated with β‐TCP or β‐TCP@CQ scaffolds. *n* = 5. Scale bar: 100 µm. (F–H) qRT‐PCR analysis of the expression of typical osteogenic markers (*opn, runx2, ocn*) in HG‐injured BMSCs treated with β‐TCP or β‐TCP@CQ scaffolds. *n* = 5. (I–K) WB analysis of Opn and Runx2 expression in HG‐injured BMSCs treated with β‐TCP or β‐TCP@CQ scaffolds. *n* = 3. (L,M) Representative immunofluorescence staining and corresponding semiquantitative analysis of Ocn (Green) and Runx2 (Red) in HG‐injured BMSCs treated with β‐TCP or β‐TCP@CQ scaffolds. Scale bar: 100 µm.

Under hyperglycemic conditions, the osteogenic differentiation capacity of BMSCs is markedly impaired, which represents a key factor contributing to the difficulty of effectively repairing diabetic bone defects. In this context, the effects of β‐TCP@CQ on activating BMSCs under HG conditions were further investigated. Notably, β‐TCP@CQ significantly reversed the decline in BMSC viability induced by the HG environment. In the HG group, cell viability decreased to approximately 70% of that in the Control group on both Day 3 and Day 7. However, treatment with β‐TCP@CQ scaffolds markedly reversed this decline and promoted substantial cell proliferation, whereas β‐TCP scaffolds had no comparable effect (Figure 4A). Regarding oxidative stress, persistent HG conditions led to excessive ROS accumulation in BMSCs, as detected by DCFH‐DA staining. However, β‐TCP@CQ significantly scavenged these elevated ROS levels, whereas β‐TCP alone did not exhibit significant ROS scavenging (Figure 4B,C).

The osteogenic differentiation capacity of HG‐injured BMSCs was further evaluated. ALP staining showed that β‐TCP@CQ reversed the inhibitory effect of HG on BMSC osteogenic differentiation (Figure 4D). Quantitative analysis revealed that ALP expression in BMSCs treated with β‐TCP@CQ was restored to levels comparable to the Control group, whereas no significant difference was observed between the β‐TCP and HG groups (Figure 4E). Moreover, qRT‐PCR confirmed that β‐TCP@CQ effectively reversed HG‐induced downregulation of key osteogenic differentiation genes, including *opn*, *runx2*, and *ocn* with fold increases of 1.1, 1.7, and 1.0, respectively (Figure 4F–H). Western blot (WB) analysis of Opn and Runx2 further verified the osteogenic inhibition caused by HG and confirmed that β‐TCP@CQ markedly increased protein expression levels compared with the HG group, whereas β‐TCP alone had no such effect (Figure 4I–K). In addition, immunofluorescence staining demonstrated the osteogenic activation effect of β‐TCP@CQ on Ocn and Runx2 expression. In HG‐injured BMSCs treated with β‐TCP@CQ, stronger green fluorescence for Ocn and red fluorescence for Runx2 were observed, with intensities comparable to those in the Control group. In contrast, the HG and β‐TCP groups exhibited weak fluorescence signals (Figure 4L,M).

### 2.5 Transcriptomic Profiling and Mechanistic Validation

**FIGURE 5:**
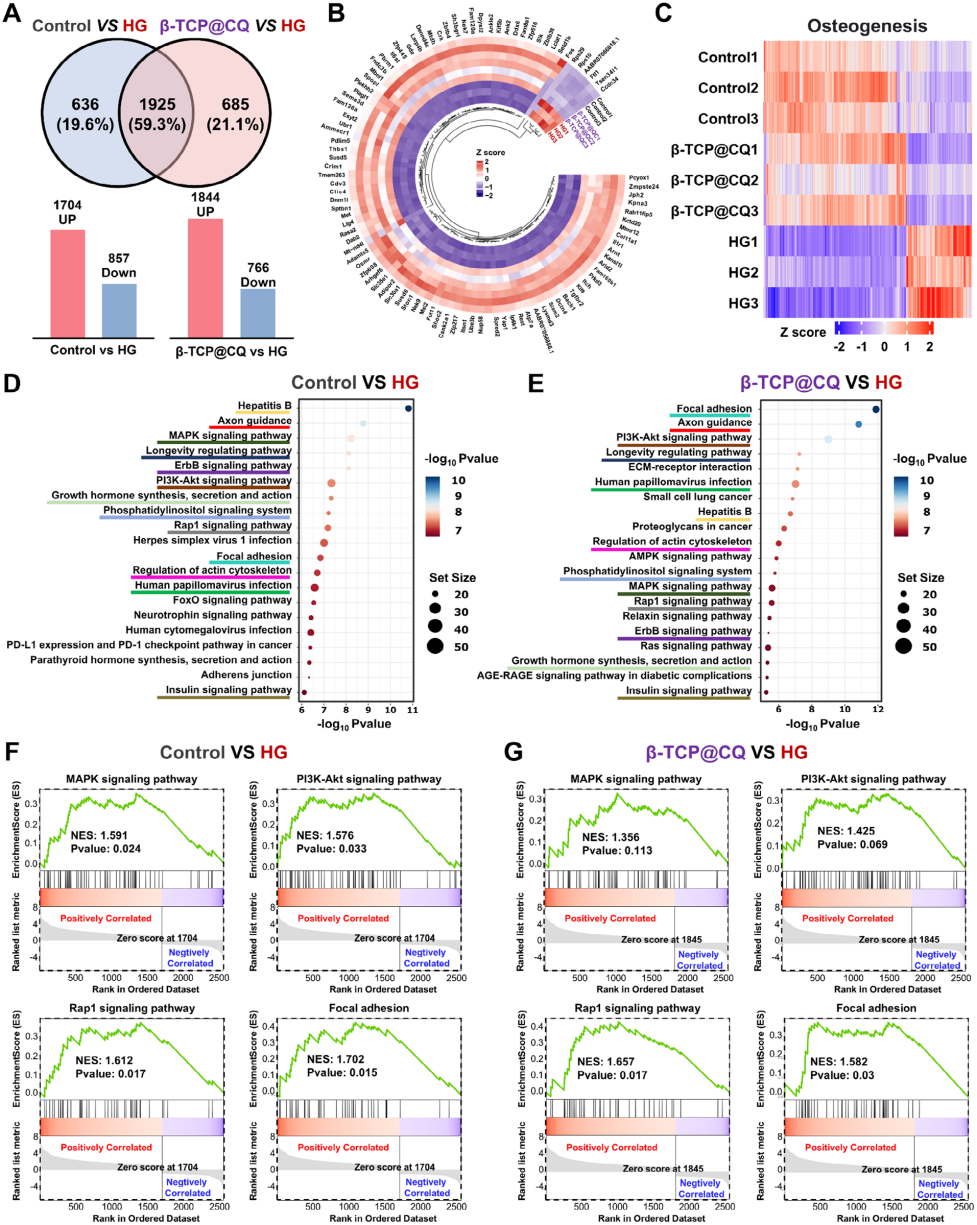
Transcriptome sequencing analysis of the biological mechanisms underlying the bioactivity of β‐TCP@CQ scaffolds. (A) Venn diagram analysis of differentially expressed genes (DEGs) between the Control group and HG group, as well as between the β‐TCP@CQ group and HG group. (B) Heatmap analysis of the top 100 most significantly DEGs. (C) Heatmap analysis of genes significantly enriched in osteogenesis‐related functions. (D) Kyoto Encyclopedia of Genes and Genomes (KEGG) pathway enrichment analysis of the top 20 upregulated signaling pathways of DEGs between the Control and HG groups. (E) KEGG pathway enrichment analysis of the top 20 upregulated signaling pathways of DEGs between the β‐TCP@CQ and HG groups. Pathways marked with the same color underline in (D) and (E) indicate common pathways. (F) Gene Set Enrichment Analysis (GSEA) of the MAPK, PI3K‐Akt, Rap1, and Focal adhesion signaling pathways between the Control and HG groups. (G) GSEA of the MAPK, PI3K‐Akt, Rap1, and Focal adhesion signaling pathways between the β‐TCP@CQ and HG groups.

Further mechanistic insights into the promotive role of β‐TCP@CQ in BMSC osteogenic differentiation under HG conditions were obtained through RNA sequencing of samples from the Control, HG injury, and β‐TCP@CQ intervention groups. Compared with the HG group, the Control and β‐TCP@CQ groups significantly upregulated 1704 and 1844 genes and downregulated 857 and 766 genes, respectively (Figure 5A). Notably, the overlap ratio of differentially expressed genes (DEGs) reached 59.5%. Heatmap analysis of the top 100 most significantly altered genes revealed that the expression profiles of the Control and β‐TCP@CQ groups were highly similar but clearly distinct from those of the HG group, suggesting that β‐TCP@CQ treatment partially reversed the detrimental effects of HG on BMSCs (Figure 5B). Consistent trends were also observed in the heatmaps enriched for “Hyperglycemia,” “Osteogenesis,” and “Angiogenesis”‐related genes (Figure 5C and Figures S14 and S15), further confirming that β‐TCP@CQ alleviated the inhibitory effects of HG osteogenic and angiogenic functions. KEGG pathway enrichment of DEGs in both Control versus HG (Figure 5D) and β‐TCP@CQ versus HG (Figure 5E) revealed that 13 of the top 20 upregulated signaling pathways overlapped (highlighted with underlines of the same color). These included well‐established pathways such as the “MAPK signaling pathway” and the “PI3K‐Akt signaling pathway,” both of which are known to regulate BMSC proliferation, survival, differentiation, and stress responses during osteogenic differentiation. Cytoskeleton‐related pathways, including the “Rap1 signaling pathway,” “Focal adhesion,” and “Regulation of actin cytoskeleton” were also enriched, indicating that β‐TCP@CQ may regulate cytoskeletal organization and morphology. In addition, pathways related to growth hormone (“Growth hormone synthesis, secretion, and action”), neural regulation (“Axon guidance”), and aging (“Longevity regulating pathway”) were also implicated in HG‐induced damage and β‐TCP@CQ‐mediated intervention. GSEA further confirmed that these signaling pathways were significantly suppressed under HG conditions but were markedly upregulated following β‐TCP@CQ treatment (Figure 5F,G and Figures S16 and S17).

**FIGURE 6:**
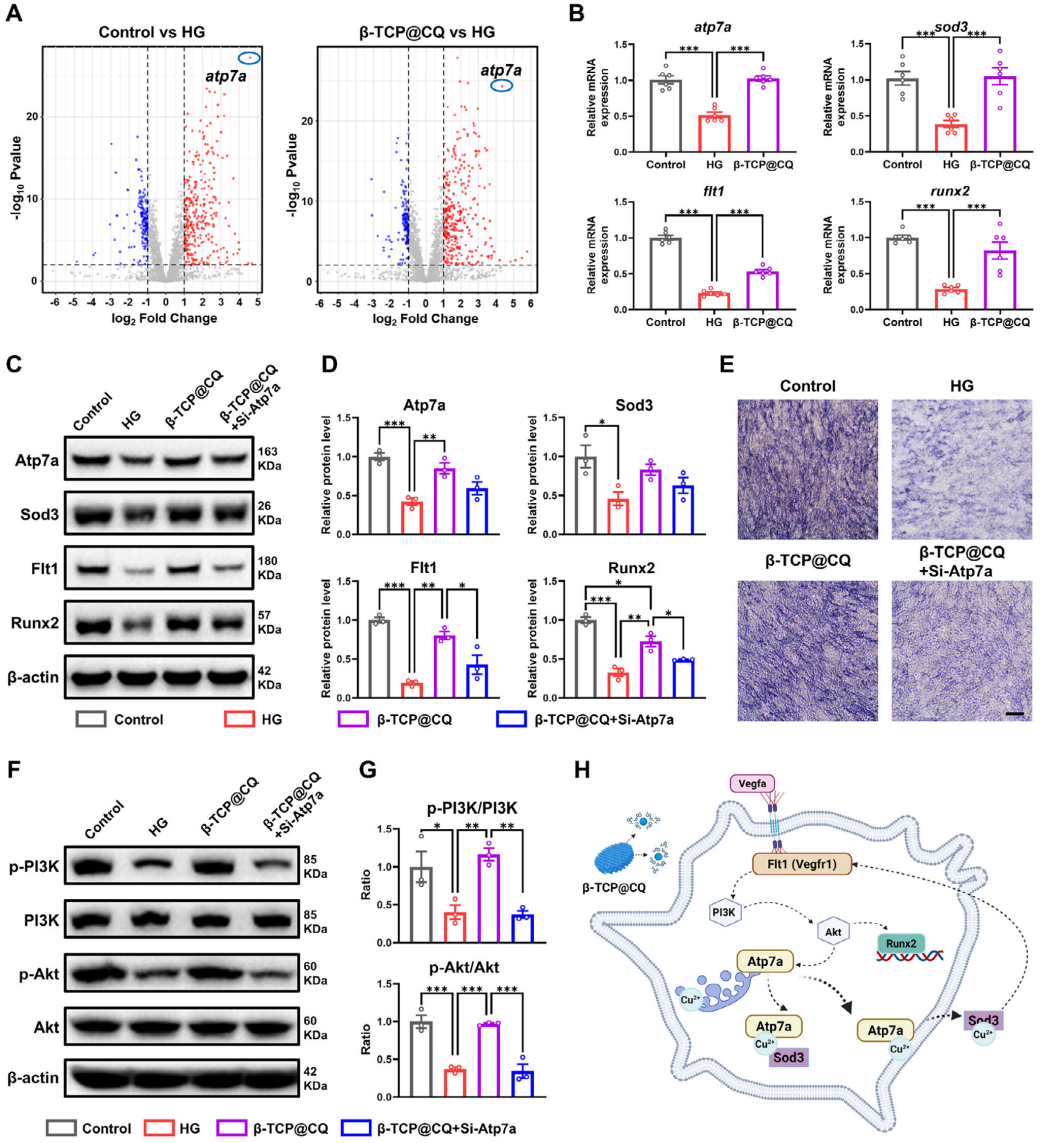
Screening and validation of key targets regulated by the β‐TCP@CQ. (A) Volcano plot analysis for the identification of key genes whose expression is reversed by β‐TCP@CQ in mitigating HG‐induced damage and osteogenic suppression. (B) qRT‐PCR analysis of the expression levels of *atp7a*, *sod3*, *flt1*, *and runx2* in HG injured BMSCs following treatment with β‐TCP@CQ. *n* = 5. (C,D) WB analysis of the expression levels of Atp7a, Sod3, Flt1, and Runx2 in HG‐injured BMSCs treated with β‐TCP@CQ in the presence of ATP7A knockdown mediated by siRNA. *n* = 3. (E) Evaluation of ALP expression in HG‐injured BMSCs treated with β‐TCP@CQ in the presence of Atp7a knockdown mediated by siRNA. Scale bar: 100 µm. (F,G) WB analysis of the regulatory effect of β‐TCP@CQ on the PI3K‐Akt pathway in HG‐injured BMSCs with Atp7a knockdown via siRNA. *n* = 3. (H) Schematic diagram illustrating the mechanism by which β‐TCP@CQ regulates osteogenesis in BMSCs under HG‐induced injury.

To identify the key genes and pathways by which β‐TCP@CQ reverses HG‐induced injury and osteogenic inhibition, a volcano plot analysis was performed (Figure 6A). Atp7a emerged as a key candidate with both high fold change and strong statistical significance. Atp7a encodes a Cu‐ATPase transport protein that is essential for maintaining cellular copper homeostasis and transports copper ions from the cytoplasm into the trans‐Golgi network lumen, thereby activating copper‐dependent enzymes in the secretory pathway, including superoxide dismutase 3 (Sod3). Previous studies have demonstrated that Sod3 regulates the vascular endothelial growth factor receptor Flt1 (Vegfr1), thereby influencing bone metabolism through the PI3K‐Akt pathway [24]. Based on this rationale, the mRNA expression levels of *atp7a*, *sod3*, *flt1*, and *runx2* were examined (Figure 6B). HG treatment significantly suppressed their expression, whereas β‐TCP@CQ markedly restored it. Similar trends were confirmed at the protein level by WB (Figure 6C,D). Furthermore, to validate the functional role of Atp7a, siRNA‐mediated knockdown was performed, which led to a significant reduction in the expression of Atp7a, Sod3, Flt1, and Runx2 in the β‐TCP@CQ group, suggesting that Atp7a may serve as a key molecular target through which β‐TCP@CQ orchestrates its regulatory effects (Figure 6C,D). ALP staining further confirmed that the pro‐osteogenic differentiation of BMSCs induced by β‐TCP@CQ was markedly impaired upon Atp7a silencing (Figure 6E and Figure S18). Moreover, phosphorylation assays of the PI3K‐Akt pathway demonstrated that β‐TCP@CQ significantly restored PI3K and Akt phosphorylation suppressed by HG, whereas Atp7a knockdown abolished this activation (Figure 6F,G). Collectively, these findings suggest that β‐TCP@CQ enhances osteogenesis under HG conditions by regulating copper homeostasis. Mechanistically, β‐TCP@CQ appears to activate Atp7a, promote copper efflux, and stimulate Sod3, which subsequently activates the FLT1‐PI3K‐Akt pathway to drive osteogenic differentiation (Figure 6H).

### 2.6 CQ Coating Enhanced the Therapeutic Efficacy of β‐TCP for Bone Defect Repair in Diabetic Rats

**FIGURE 7:**
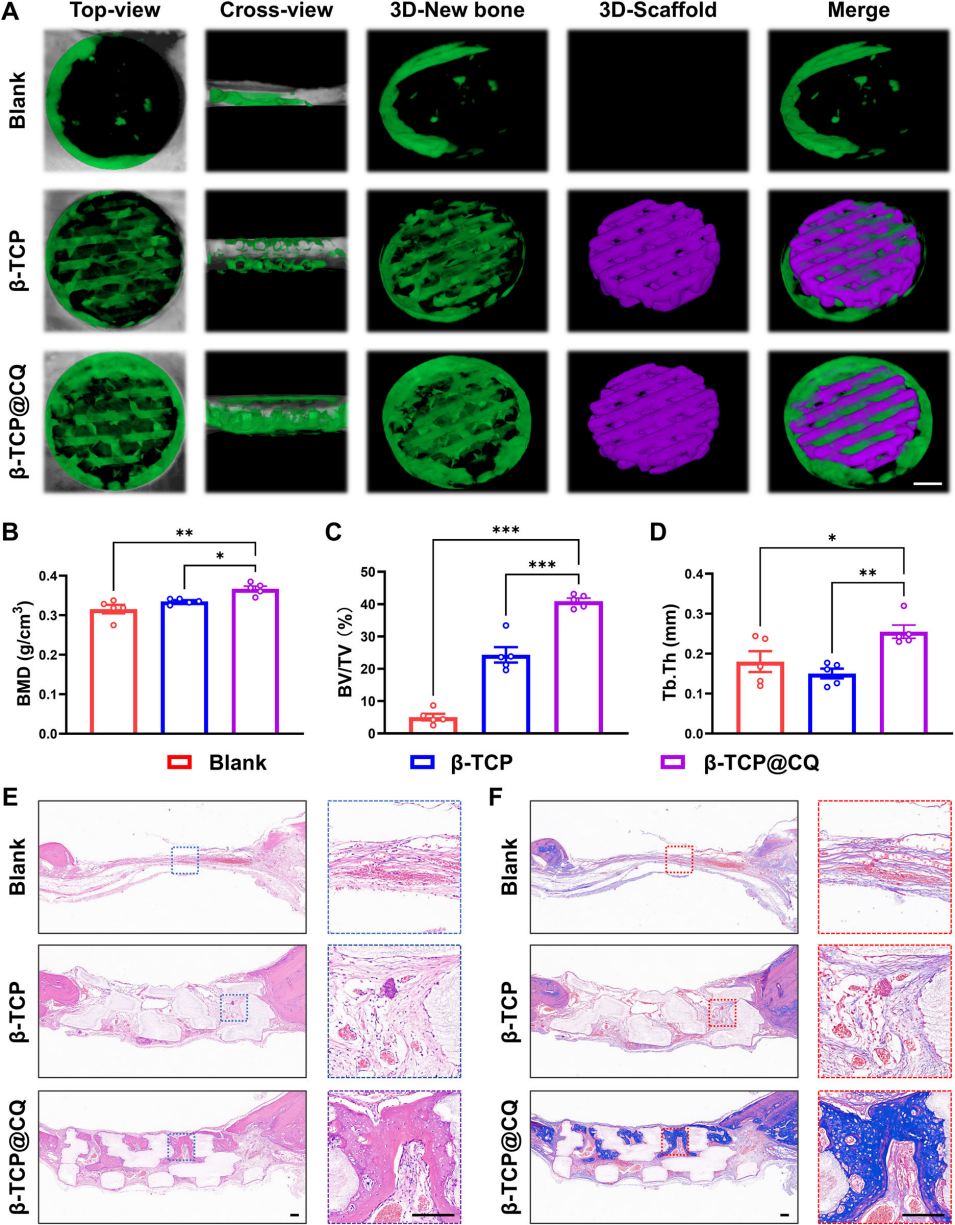
In vivo bone generation of β‐TCP@CQ in diabetic rat. (A) Representative micro‐computed tomography (CT) images showing 3D reconstructions of the defect areas in diabetic cranial bone defects treated with β‐TCP or β‐TCP@CQ. Newly formed bone is shown in green, and the implanted scaffold is shown in purple. Scale bar: 1 mm. (B–D) Quantitative analysis of bone regeneration parameters within the defect area based on micro‐CT data, including bone mineral density (BMD), bone volume fraction (BV/TV), and trabecular thickness (Tb.Th). *n* = 5. (E,F) Representative histological images of hematoxylin and eosin (H&E) staining and Masson's trichrome staining of diabetic bone defects treated with β‐TCP or β‐TCP@CQ. Scale bar: 200 µm.

After confirming that β‐TCP@CQ promotes osteogenic differentiation of BMSCs and angiogenic differentiation of HUVECs under HG conditions, its potential to facilitate diabetic bone defect repair in vivo was further investigated. A cranial defect model was established in type 2 diabetic (T2DM) rats. Successful establishment of the T2DM rat model was verified using the intraperitoneal glucose tolerance test (IPGTT) and intraperitoneal insulin tolerance test (IPITT). As shown in Figure S19, the sustained hyperglycemic state in the T2DM group confirmed successful model establishment. Furthermore, IPITT analysis demonstrated an insulin‐resistant phenotype in the T2DM group, with significantly impaired insulin sensitivity compared with the Control group. Following scaffold implantation into the cranial defect sites, bone repair was evaluated after 8 weeks. Bone regeneration was assessed by micro‐CT. In the Blank group, minimal new bone formation was observed at the periphery of the defect. In contrast, both β‐TCP and β‐TCP@CQ promoted new bone formation within the defect, with β‐TCP@CQ exhibiting significantly superior osteogenic effects compared with β‐TCP (Figure 7A). Quantitative micro‐CT analysis confirmed the superior osteogenic performance of β‐TCP@CQ. Bone mineral density (BMD), bone volume fraction (BV/TV), and trabecular thickness (Tb.Th) in the β‐TCP@CQ group were significantly higher than those in both the β‐TCP and Blank groups. Specifically, BMD, BV/TV, and Tb.Th in the β‐TCP@CQ group were 1.07‐, 1.68‐, and 1.69‐fold higher, respectively, than in the β‐TCP group. Notably, although the BV/TV value in the β‐TCP group was significantly higher than in the Blank group, no significant differences were observed in BMD or Tb.Th, indicating that the newly formed bone in the β‐TCP group was of inferior quality (Figure 7B–D).

**FIGURE 8:**
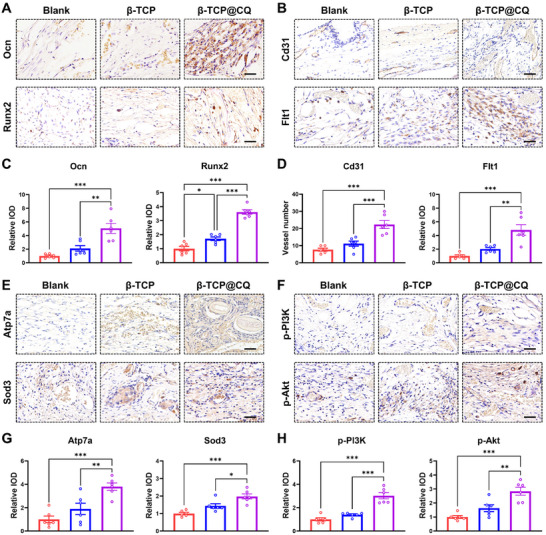
Immunohistochemical analysis validating the promoting effect of β‐TCP@CQ on diabetic bone regeneration and its underlying mechanisms. (A) Immunohistochemical staining for Ocn and Runx2 to evaluate the osteogenic potential of β‐TCP@CQ. Scale bar: 25 µm. (B) Immunohistochemical staining for Cd31 and Flt1 to evaluate the angiogenic potential of β‐TCP@CQ. Scale bar: 25 µm. (C) Quantitative statistical analysis of the expression levels of Ocn and Runx2. *n* = 6. (D) Quantitative statistical analysis of the expression levels of Cd31 and Flt1. *n* = 6. (E) Immunohistochemical staining to investigate the activation of Atp7a and Sod3 by β‐TCP@CQ. Scale bar: 25 µm. (F) Immunohistochemical staining of p‐PI3K and p‐Akt to explore the activation of the PI3K‐Akt signaling pathway by β‐TCP@CQ. Scale bar: 25 µm. (G,H) Quantitative statistical analysis of the expression levels of Atp7a, Sod3, p‐PI3K, and p‐Akt. *n* = 6.

Histological analyses were then performed to evaluate the quality of newly formed bone. Hematoxylin and eosin (H&E) staining revealed severely impaired bone healing in the diabetic condition, characterized by limited new bone formation and extensive fibrous tissue infiltration. In contrast, the β‐TCP@CQ group showed markedly reduced inflammatory infiltration and abundant new bone formation (Figure 7E). Quantitative analysis confirmed that the new bone area was significantly greater in the β‐TCP@CQ group than in both the β‐TCP and Blank groups (Figure S20). Masson's trichrome staining further demonstrated enhanced collagen deposition in the β‐TCP@CQ group, which was quantitatively greater than in the other groups (Figure 7F and Figure S21). Immunohistochemical staining was performed to examine osteogenic and angiogenic markers, including Ocn, Runx2, Cd31, and Flt1. β‐TCP@CQ markedly increased expression of all four markers in diabetic bone defects (Figure 8A,B). Quantitative analysis showed that β‐TCP induced only modest increases, whereas β‐TCP@CQ substantially upregulated their expression, consistent with its superior osteogenic and angiogenic functions (Figure 8C,D). In addition, β‐TCP@CQ activated Atp7a, Sof3, and the PI3K‐Akt pathway in vivo, as revealed by immunohistochemistry (Figure 8E,F). Quantitative analysis further confirmed that expression levels were significantly higher in the β‐TCP@CQ group than in either the β‐TCP or Blank groups (Figure 8G,H). Collectively, these findings demonstrate that β‐TCP@CQ promotes bone defect repair under diabetic conditions by regulating copper homeostasis. Specifically, the coating activates ATP7A, facilitates copper efflux, stimulates SOD3, and subsequently activates the FLT1/PI3K‐Akt signaling pathway, thereby driving osteogenesis and angiogenesis.

## 3 Discussion

A wide range of bone repair materials have been adopted in clinical practice, including metallic alloys (e.g., Ti), ceramic scaffolds (e.g., β‐TCP), and polymeric matrices (e.g., PCL) [25]. These materials differ in chemical composition, mechanical strength, and degradation profiles, thereby providing clinicians with multiple options for repairing bone defects and achieving notable success under normal physiological conditions. However, under pathological conditions such as diabetes, the regenerative potential of bone tissue is markedly impaired, and the pro‐healing efficacy of conventional materials is substantially compromised [26]. Enhancing the functional adaptability of existing implants to diabetic conditions has therefore emerged as a critical challenge in orthopedic biomaterials research. In this study, a surface functionalization strategy based on metal–flavonoid nanocoatings was proposed. Among the tested combinations, the CQ coating was identified as the optimal candidate, exhibiting multifunctional properties including antioxidant activity, osteogenic promotion, and angiogenic induction. When applied to β‐TCP, a conventional bone repair material, this coating significantly enhanced bone regeneration under diabetic conditions.

The microenvironmental disorder in diabetic bone defects is a complex process involving multiple factors, including ROS‐mediated oxidative stress, impaired bone regeneration, and defective angiogenesis [8]. These pathological features are closely associated with ionic microenvironmental imbalance, collectively forming a vicious cycle. Specifically, hyperglycemia accelerates nonenzymatic protein glycation, generating AGEs, which disrupt mitochondrial electron transport and activate NADPH oxidase, thereby triggering excessive ROS production [8]. ROS can directly oxidize cysteine residues on ion transporters, forming disulfide bonds that alter protein conformation and impair ion binding and transport [27]. Consequently, many ion transporters, including TRPM6/TRPM7 and ATP7A/ATP7B, are inactivated or downregulated [28]. Given that many metal ions (e.g., Mg^2+^, Co^2+^, Cu^2+^, Zn^2+^, Sr^2+^) play indispensable roles in bone defect repair by regulating proliferation, differentiation, matrix synthesis, and mineralization, ionic imbalance may impair bone regeneration in diabetes [29, 30, 31]. Although implant materials capable of modulating the ionic microenvironment have been developed, such as biodegradable metals, ceramics, and organic–inorganic hybrid hydrogels, their compositions are relatively fixed to meet requirements for strength and biocompatibility [32]. Furthermore, degradation of these materials may be influenced by the fluctuating diabetic metabolic microenvironment, leading to uncontrollable ion release and unstable therapeutic efficacy [26, 33]. In contrast, this study exploited the self‐assembly properties of metal–flavonoid complexes to construct a coating capable of stabilizing metal ions on virtually any substrate. Flavonoid compounds provided abundant phenolic hydroxyl and carbonyl groups to form strong chemical bonds with material surfaces, whereas chelation with metal ions anchored inorganic ions around implants, thereby maintaining a stable ionic microenvironment [11, 34]. Based on this property, a multifunctional library of self‐assembled metal–flavonoid coatings was established, providing diverse options for treating diabetic bone defects. Moreover, flavonoids inherently possess antioxidant activity, which synergizes with metal ions to further enhance therapeutic efficacy.

In the treatment of diabetic bone defects, impaired osteogenesis and angiogenesis represent critical challenges that urgently need to be addressed. Therefore, screening the optimal osteogenic and angiogenic formulation from the metal–flavonoid coating sample library holds significant importance for improving therapeutic outcomes. Among various combinations (Mg, Co, Cu, Zn, and Sr combined with luteolin, curcumin, or quercetin), CQ demonstrated superior osteogenic and angiogenic activities. While most coatings exhibited osteogenic effects comparable to CQ, only Cu‐containing coatings (Cu‐luteolin and Cu‐curcumin) promoted angiogenesis to a similar extent. Considering that Cu ions are known to tightly bind vascular growth factors such as VEGF, bFGF, and HIF‐1α, stabilizing their structures and enhancing their activities, it is not surprising that Cu‐containing combinations exhibited the strongest angiogenic capabilities [35]. Furthermore, CQ coatings exhibited a more balanced and sustained effect than other pro‐angiogenic strategies. Historically, to compensate for insufficient angiogenesis in diabetes, local administration of recombinant growth factors or gene therapy to promote VEGF overexpression has been attempted [36, 37]. Although these methods can increase local vessel density in the short term, they present limitations including short duration, challenging dose control, and potential aberrant neovascularization. By contrast, copper, an essential trace element, has a broader therapeutic window [38], and its release can be modulated through quercetin coordination, thereby reducing the risk of toxicity. In this system, quercetin scavenged hyperglycemia‐induced ROS, reducing endothelial inflammation and cell death, while copper enhanced angiogenic signaling. This dual action promoted timely microvessel ingrowth into defects, as evidenced by early CD31‐positive vessel formation and improved new bone quality in diabetic rat cranial defect models.

Importantly, ATP7A was identified as a key mediator of CQ function. Under hyperglycemic conditions, ATP7A expression in BMSCs was markedly suppressed, whereas CQ restored ATP7A expression. This effect was attributed to the sustained Cu‐rich microenvironment created by CQ, which may protect cysteine residues in ATP7A from AGE‐induced damage, thereby preserving transporter function. This change triggers a cascade of cellular signaling pathways and physiological processes, indirectly influencing multiple effectors and ultimately achieving antioxidation, osteogenesis, and angiogenesis. Specifically, upon ATP7A activation, copper‐transport function is restored, facilitating the transfer of Cu ions to organelles such as the Golgi apparatus [39]. This process ensures proper incorporation of copper into SOD3 during its synthesis, thereby maintaining normal enzymatic activity and converting active superoxide anions (•O_2_^−^) into H_2_O_2_ and O_2_, thus reducing oxidative‐stress‐induced cellular damage [40]. Additionally, restored SOD3, by regulating the intracellular redox state, significantly modulates FLT1 expression and function, thereby promoting endothelial cell proliferation, migration, and lumen formation [24, 41]. FLT1 activation further triggers PI3K, which catalyzes the conversion of phosphatidylinositol‐4,5‐bisphosphate (PIP_2_) into phosphatidylinositol‐3,4,5‐trisphosphate (PIP_3_) [24, 41]. Acting as a second messenger, PIP_3_ recruits Akt to the plasma membrane and promotes its phosphorylation and activation [24, 41]. Activated Akt, in turn, promotes osteoblast proliferation and differentiation while inhibiting osteoclast activity, thereby supporting bone regeneration [24, 41].

Functionally, CQ treatment markedly upregulated ALP activity, osteogenic marker genes (OPN, OCN, RUNX2) and proteins (OCN, RUNX2, etc.) in BMSCs compared with the untreated HG group, approaching levels observed under normoglycemic conditions. In contrast, when only general antioxidants (e.g., N‐acetylcysteine, NAC) or SOD mimics (e.g., Tempol) were administered, ROS was only partially reduced [42, 43], but endogenous mechanisms such as ATP7A‐SOD3 could not be restored, and improvement of osteogenic function was therefore relatively limited. Additionally, materials directly delivering growth factors such as bone morphogenetic proteins (BMPs) have been used to promote osteogenesis in diabetes; however, oxidative stress and AGEs in hyperglycemic environments can markedly attenuate the efficacy of exogenous factors, and BMPs may cause adverse effects, such as ectopic ossification [44]. By contrast, the CQ coating achieves effects by mobilizing endogenous osteogenic differentiation programs, such as activating PI3K‐Akt, which is more physiological and robust. In addition, ATP7A was recently reported to limit autophagic degradation of VEGFR2 in endothelial cells, suggesting that copper homeostasis is also important for maintaining the integrity of vascular signaling pathways [45]. Therefore, coatings that restore ATP7A function may help maintain VEGFR2 levels and improve endothelial responses to pro‐angiogenic stimulation. This advantage extends beyond methods that simply supply exogenous factors. Overall, by alleviating oxidative stress and re‐engaging cellular signaling, the CQ coating is more effective than most single‐modality interventions at improving the osteogenic differentiation capacity of BMSCs under diabetic conditions.

From a clinical perspective, metal–flavonoid coatings offer distinct advantages but also pose challenges. In terms of advantages, their multifunctionality enables simultaneous mitigation of oxidative stress, osteogenic deficiency, and angiogenic impairment, thereby reducing the need for multiple interventions. They are also cost‐effective, widely accessible, and applicable via a simple one‐step coating method, facilitating large‐scale production and broad compatibility with diverse implants. Moreover, as members of the broader polyphenol family, many other polyphenolic compounds, such as phenolic acids (e.g., gallic acid), tannins (e.g., tannic acid), and catechins (e.g., tea polyphenols), also exhibit strong metal–chelating capabilities and distinct biological activities [46, 47, 48]. These polyphenols may share similar behavior during metal–polyphenol self‐assembly and could likewise generate functional coatings on conventional bone repair materials, thereby providing additional options for coating design. However, potential limitations and areas for further research must also be acknowledged. First, the dose of released copper ions requires strict control. Balancing biological efficacy with avoidance of cytotoxicity is crucial in material design, as excessive copper release can lead to high local concentrations that cause cellular stress or systemic copper overload [49]. Second, the in vivo stability and metabolism of quercetin must be considered. Although chelation enhances and stabilizes its antioxidant capacity, long‐term presence in the body may result in metabolic clearance or biotransformation, necessitating evaluation of its effective duration in vivo. Furthermore, although CQ exhibited the best osteogenic and angiogenic functionalities within the constructed metal–flavonoid coating library, the scope of screening remained limited. Expanding the coating library and adopting new screening approaches (e.g., machine learning) will be necessary to provide a stronger data foundation for subsequent clinical translation. Overall, there is justified optimism regarding the clinical application of metal–flavonoid coatings for repairing diabetic bone defects. The platform is modular and could incorporate other agents (e.g., insulin, metformin) to form more comprehensive therapeutic implants. Additionally, this strategy could be extended to other bone conditions characterized by oxidative stress and impaired healing, such as fractures in older adults and postinfection reconstruction. In the field of regenerative medicine, the concept of metal–flavonoid coatings exemplifies a novel approach that combines natural products with inorganic elements to achieve multifunctional synergy. With the convergence of materials science and biomedical research, such multifunctional materials are poised to play an increasingly important role in clinical bone tissue engineering.

## 4 Conclusion

In this study, a metal–flavonoid functionalization strategy for implant surfaces was established. The approach rationally integrates osteogenic and angiogenic metal ions with natural antioxidant flavonoids, enabling one‐pot construction of a versatile bioactive coating. The strategy proved broadly applicable to diverse bone repair substrates, including metals, ceramics, polymers, and their composites, as well as to diverse structural forms, such as 2D surfaces and 3D scaffolds. By imparting defined biological functions, the coating modulates the local defect microenvironment and thereby enhances the regenerative capacity of conventional materials under pathological conditions. As a representative implementation, a copper‐quercetin (CQ) coating was developed and optimized to augment the performance of β‐TCP in diabetic bone healing. Mechanistically, CQ promoted osteogenesis and angiogenesis by regulating copper homeostasis and activating the PI3K‐Akt pathway. Collectively, metal–flavonoid functionalization provides a mechanistically grounded, readily deployable platform for expanding the therapeutic potential of established bone‐repair materials in complex pathological settings.

## 5 Experimental Section

### 5.1 Materials

Luteolin, quercetin, curcumin, magnesium chloride, cobalt chloride, copper chloride, zinc chloride, strontium chloride, and sodium hydroxide (NaOH) were purchased from Macklin (Shanghai, China). Titanium was obtained from Changzhou Bokang Special Material Technology Co., Ltd. (Changzhou, China), and 316L stainless steel (sheet and strip) was purchased from Ningbo Qihong Stainless Steel Co., Ltd. (Ningbo, China). Polycaprolactone (PCL) was obtained from DaiGang Biology (Shandong, China), and polyether ether ketone (PEEK) was obtained from Jilin Zhongyan Polymer Materials Co., Ltd. (Jilin, China). β‐Tricalcium phosphate (β‐TCP) and calcium silicate (CS) powders were purchased from Kunshan Chinese Technology New Materials Co., Ltd. (Jiangsu, China). Osstem dental implants were purchased from Osstem Implant Co., Ltd. (Busan, South Korea). The Cell Counting Kit‐8 (CCK‐8) was purchased from Yeasen Biotechnology Co., Ltd. (Shanghai, China). Antibodies against Runt‐related transcription factor 2 (RUNX2), vascular endothelial growth factor receptor 1 (FLT1), cluster of differentiation 31 (CD31), and superoxide dismutase 3 (SOD3) were obtained from Affinity Biosciences (Cincinnati, OH). Antibodies against ATPase copper‐transporting alpha (ATP7A), osteopontin (OPN), and osteocalcin (OCN) were purchased from Abclonal Technology (Wuhan, China). Antibodies against phosphorylated phosphoinositide 3‐kinase (p‐PI3K) and phosphorylated protein kinase B (p‐Akt) were obtained from Abways Technology (Shanghai, China). Hydroxyl radical, superoxide anion, and hydrogen peroxide assay kits were purchased from Solarbio Science & Technology Co., Ltd. (Beijing, China). Fetal bovine serum (FBS) and polymerase chain reaction (PCR) reagents were obtained from Vazyme Biotech Co., Ltd. (Nanjing, China).

### 5.2 Preparation of Base Materials

Ti, 316L, and PEEK discs were purchased directly from the manufacturers. PCL discs were prepared by melting PCL at 80°C and then mold‐casting. CS and β‐TCP discs were prepared by uniaxial pressing of the powders with a small amount of polyvinyl alcohol (PVA) as a binder at 200 MPa, followed by sintering at 1000 and 1100°C for 3 h, respectively. PCL/β‐TCP discs were fabricated by mixing β‐TCP powder (10 wt%) into the PCL matrix at 80°C, followed by mold‐casting. Porous β‐TCP scaffolds were fabricated using a stereolithography‐based 3D printing technique. Briefly, β‐TCP powder was mixed with a photosensitive resin at a 1:1 ratio to prepare the printing ink, which was then loaded into the resin tank of a stereolithography‐based 3D printer. The printing was carried out under ultraviolet (UV) irradiation at 10 mW/cm^2^ for 15 s per layer until the construct was completed. The printed green bodies were subsequently sintered at 1100°C to remove the resin and induce powder recrystallization, yielding β‐TCP scaffolds.

### 5.3 Preparation of Metal–Flavonoid Coatings

The copper‐quercetin (CQ) coating was prepared as a representative example. A 50 mL quercetin aqueous solution (0.06 mol/L, solution A) was prepared and adjusted to pH 10 with NaOH. Separately, a 50 mL copper chloride solution (0.03 mol/L, solution B) was prepared. Substrates were placed into 5 mL round‐bottom centrifuge tubes, followed by addition of 1.5 mL solution A and 1.5 mL solution B. The mixture was shaken at 100 rpm for 3 h. The resulting coatings were rinsed with deionized water and freeze‐dried to obtain scaffolds with the desired coating. Other metal–flavonoid coatings were prepared using the same procedure with corresponding flavonoid and metal chloride precursors.

### 5.4 Screening of Metal–Flavonoid Coatings

The osteogenic and angiogenic activities of BMSCs and HUVECs under HG conditions were evaluated to screen different metal–flavonoid coatings. Specifically, BMSCs (isolated from neonatal rat bone marrow) were seeded onto various metal–flavonoid coated scaffolds in 24‐well plates at 2 × 10^4^ cells per well. After attachment, the medium was replaced with HG (50 mm) medium supplemented with osteogenic induction factors, and the cells were cultured for 7 days. ALP activity was quantitatively determined using a commercial kit (Beyotime, Beijing, China) with p‐nitrophenyl phosphate as the chromogenic substrate. ALP activity was normalized to total cellular protein measured by a bicinchoninic acid (BCA) assay. For the tube formation assay, HUVECs (purchased from the Shanghai Cell Bank of the Chinese Academy of Sciences) were seeded on different metal–flavonoid coated scaffolds in 24‐well plates at a density of 5 × 10^4^ cells/well. After attachment, the medium was replaced with HG medium and the cells were cultured for 3 days, followed by enzymatic detachment for subsequent tube formation experiments. Matrigel (ABW, China) was thawed on ice at 4°C and added to pre‐cooled 48‐well plates, which were then incubated at 37°C for 30 min to allow gelation. A suspension containing HUVECs were seeded onto the Matrigel‐coated wells at a density of 4 × 10^4^ cells/well. The cells were incubated at 37°C for 6 h, imaged by microscope, and total tube length was quantified using ImageJ software (National Institutes of Health, USA).

### 5.5 MD Simulations

MD simulations were performed using the Forcite module in Materials Studio. The molecular structures of quercetin and Cu^2+^ were constructed and subjected to geometry optimization. The simulation box was generated using the Amorphous Cell module, containing 148 quercetin molecules and 20 Cu^2+^ ions. The COMPASS III force field was applied throughout the simulations. System density at 298 K was obtained under the isothermal‐isobaric (NPT) ensemble for 500 ps, after which the system was placed on the (001) surface. Subsequently, the canonical (NVT) ensemble was applied to equilibrate the system at 298 K for 2 ns. The final 2 ns of the NVT trajectory run were used for radial distribution function (RDF) and mass density analyses.

### 5.6 Characterization of the Screened CQ Coated β‐TCP (β‐TCP@CQ)

Overall scaffold morphology was observed using an optical microscope (Olympus, Japan). Scaffold microstructures were examined by SEM (JSM‐7800F, JEOL, Japan) equipped with EDS for elemental mapping. 3D architecture and porosity were analyzed using high‐resolution micro‐computed tomography (micro‐CT; Skyscan 1276, Bruker, Belgium). Elemental valence states of β‐TCP@CQ disc were determined by X‐ray photoelectron spectroscopy (XPS; PHI 5000 VersaProbe III, ULVAC‐PHI, Japan), and crystal structures were characterized by X‐ray diffraction (XRD; D8 Advance, Bruker, Germany). UV–vis absorption spectra of quercetin and CQ solutions were recorded using a UV–Vis spectrophotometer (Cary 5000, Agilent, USA). FTIR spectra were further collected using an FTIR spectrometer (Bruker, Germany).

Scratch testing was performed on β‐TCP@CQ disc using a calibrated NanoTest system (Micro Materials Ltd., Wrexham, UK) with a diamond Rockwell conical–spherical indenter (tip radius 9 µm). During progressive load scratch testing, the applied load increased linearly to a maximum of 500 mN. Mechanical properties of the scaffolds were evaluated by uniaxial compression using a universal testing machine (Instron, USA) at a loading rate of 2 mm/min. Compressive Young's modulus was calculated from the linear region of the stress–strain curve.

For in vitro degradation and ion release studies, scaffolds were immersed in neutral Tris‐HCl buffer and collected on days 1, 3, 7, and 14, followed by drying and weighing. The degradation rate was calculated as the percentage of weight loss. Supernatants collected at each time point were analyzed to quantify released quercetin and copper ions by UV–vis spectrophotometry and inductively coupled plasma mass spectrometry (ICP‐MS; 7850, Agilent, Singapore), respectively.

### 5.7 DPPH Radical Scavenging Assay

A 1 mg/mL stock solution of DPPH was prepared and diluted 50‐fold prior to use. Scaffolds were incubated with 2 mL of the diluted DPPH solution at 37°C for 30 min. Absorbance at 517 nm of the solution was measured before and after incubation.

### 5.8 ABTS•^+^ Radical Scavenging Assay

A 7.4 mmol/L ABTS solution was prepared and mixed with an equal volume of 2.6 mmol/L potassium persulfate solution. The mixture was incubated at room temperature for 18 h to generate ABTS•^+^ radicals and subsequently diluted 50‐fold for use. Scaffolds were incubated with 2 mL of the diluted ABTS•⁺ solution at 37°C for 30 min, and absorbance at 734 nm was measured before and after incubation. The radical scavenging rate was calculated as

(1) $$\mathrm{Scavenging}\;\mathrm{rate}\left(\%\right)=\left(1-A_{\mathrm{after}}/A_{\mathrm{before}}\right)\times 100\%$$

where A_before_ and A_after_ represent the absorbance before and after incubation, respectively.

The scavenging of different ROS (H_2_O_2_, •O_2_^−^, and •OH) was measured using the corresponding commercial assay kits according to the manufacturer's instructions.

### 5.9 Effects of CQ Coatings on HG‐Injured HUVECs

Cell viability assay: HUVECs were seeded onto β‐TCP and β‐TCP@CQ scaffolds in 96‐well plates at a density of 5 × 10^3^ cells/well and incubated in HG medium. Cell viability was assessed on days 1, 3, and 7 using the Cell Counting Kit‐8 (CCK‐8, Yeasen, Shanghai, China). Briefly, culture medium was removed and replaced with fresh medium containing 10 µL CCK‐8 reagent per well. After incubation at 37°C for 1.5 h, absorbance at 450 nm was measured using a microplate reader (Epoch 2NS, BioTek, USA).

Intracellular ROS detection: HUVECs were seeded onto β‐TCP and β‐TCP@CQ scaffolds in 24‐well plates at a density of 5 × 10^4^ cells/well and cultured in HG medium for 3 days. Cells were then harvested and stained with 2′,7′‐dichlorofluorescin diacetate (DCFH‐DA; Beyotime, Shanghai, China) to assess intracellular ROS levels. Briefly, cells were incubated with DCFH‐DA in fresh medium at 37°C for 30 min, washed twice with dulbecco's modified eagle medium (DMEM), and counterstained with Hoechst (Baitg, China) for 5 min. Fluorescence images were acquired using an Operetta CLS high‐content imaging system (PerkinElmer, USA), and fluorescence intensity was quantified using ImageJ software (NIH, USA).

Scratch wound assay: HUVECs pretreated under HG conditions with different scaffolds were digested and seeded into 6‐well plates to form a confluent monolayer. A linear scratch was created across the monolayer using a 200 µL pipette tip. Images were captured immediately and after 24 h using an inverted microscope (CKX53, Olympus, Japan). Cell migration rates were quantified by ImageJ analysis with pseudocolor mapping.

Tube formation assay: HUVECs pretreated under HG conditions with different scaffolds were harvested and seeded onto Matrigel‐coated 24‐well plates (ABW, China). After incubation at 37°C for 6 h, tube‐like structures were imaged by microscope, and the number of nodes and total tube length were quantified using ImageJ software.

Quantitative real‐time PCR (qRT‐PCR): Total RNA was extracted from HUVECs cultured on scaffolds for 3 days using a commercial RNA extraction kit. Complementary DNA (cDNA) was synthesized using a SYBR Premix Kit. qRT‐PCR was performed to analyze the mRNA expression of vascular endothelial growth factor A (VEGFA), endothelial nitric oxide synthase (eNOS), and hypoxia‐inducible factor‐1α (HIF‐1α). Relative expression levels were normalized to glyceraldehyde‐3‐phosphate dehydrogenase (GAPDH) as the housekeeping gene.

### 5.10 Effects of CQ Coatings on HG‐Injured BMSCs

Cell viability assay: BMSCs were seeded onto β‐TCP and β‐TCP@CQ scaffolds in 96‐well plates at a density of 5 × 10^3^ cells/well and incubated in HG medium. Cell viability was assessed on days 1, 3, and 7 using the Cell Counting Kit‐8.

Intracellular ROS detection: BMSCs were seeded onto β‐TCP and β‐TCP@CQ scaffolds in 24‐well plates at a density of 5 × 10^4^ cells/well and cultured in HG medium for 3 days. Cells were then harvested and stained with DCFH‐DA to assess intracellular ROS.

ALP staining: BMSCs were seeded in 24‐well plates at a density of 2 × 10^4^ cells/well, and β‐TCP or β‐TCP@CQ scaffolds were placed in the upper chamber of a transwell insert. Cells were cultured for 7 days in HG medium supplemented with osteogenic induction factors. Cells were then fixed with 4% paraformaldehyde for 15 min and stained with a commercial ALP staining kit (Beyotime, Shanghai, China) for 30 min. The formation of purple precipitates was visualized under a light microscope (CKX53; Olympus, Japan).

qRT‐PCR: BMSCs were seeded onto β‐TCP and β‐TCP@CQ scaffolds in 12‐well plates at a density of 5 × 10^4^ cells/well and cultured in HG medium supplemented with osteogenic induction factors for 7 or 14 days. Total RNA was extracted and reverse‐transcribed into cDNA, which was used as the template for PCR amplification with specific primers to quantify the mRNA expression of osteopontin (OPN), Runt‐related transcription factor 2 (RUNX2), and osteocalcin (OCN). Relative gene expression levels were normalized to GAPDH.

Western blot (WB): BMSCs subjected to HG stimulation and scaffold treatment were lysed on ice for 30 min with RIPA buffer supplemented with protease inhibitors. Total protein concentration was determined using a BCA assay kit. Prior to electrophoresis, protein samples were mixed with 4× Laemmli buffer and boiled at 95°C for 10 min. Proteins were separated by sodium dodecyl sulfate‐polyacrylamide gel electrophoresis (SDS‐PAGE) and transferred onto 0.22 µm PVDF membranes (Millipore, USA) using wet transfer. Membranes were blocked with 5% bovine serum albumin (BSA) in TBST for 2 h at room temperature, followed by overnight incubation at 4°C with primary antibodies against OPN and RUNX2. After washing, membranes were incubated with horseradish peroxidase (HRP)‐conjugated secondary antibodies for 1 h at room temperature. Protein bands were visualized using a chemiluminescence imaging system, and band intensities were quantified by ImageJ software with normalization to β‐actin.

Immunofluorescence staining: BMSCs were seeded in 24‐well plates at a density of 1 × 10^4^ cells/well, and β‐TCP or β‐TCP@CQ scaffolds were placed in the upper chamber of a transwell insert. Cells were cultured for 7 days (for RUNX2 staining) or 14 days (for OCN staining) in HG medium containing osteogenic induction factors. Cells were fixed with 4% paraformaldehyde for 20 min and washed three times with PBS. Permeabilization was performed with 0.1% Triton X‐100 in PBS for 20 min, followed by blocking with 5% BSA for 1 h at room temperature. Cells were incubated overnight at 4°C with primary antibodies against RUNX2 or OCN, followed by incubation with fluorescently labeled secondary antibodies for 2 h at room temperature. Samples were mounted with an antifade medium containing DAPI. Fluorescent images were acquired using a laser scanning confocal microscope (CSU‐W1 SoRa; Nikon, Germany), and fluorescence intensity was quantified with ImageJ software.

RNA sequencing (RNA‐seq): Total RNA from cells cultured in normal medium, HG medium, and HG medium supplemented with β‐TCP@CQ scaffolds for 14 days was extracted using TRIzol reagent according to the manufacturer's protocol. RNA concentration and purity were determined using a NanoDrop 2000 spectrophotometer (Thermo Scientific), and RNA integrity was assessed on an Agilent 2100 Bioanalyzer (Agilent Technologies) with RNA Integrity Number (RIN) > 8.0. RNA‐seq library preparation and sequencing were performed by Lianchuan Bio (Hangzhou, China). Briefly, poly(A)‐containing mRNA was enriched from total RNA using oligo(dT) magnetic beads. mRNA was then fragmented into short segments, reverse‐transcribed into cDNA, and subjected to end repair, 3'‐end adenylation, and adapter ligation. After size selection and PCR amplification, libraries were sequenced on an Illumina NovaSeq 6000 platform in a 150 bp paired‐end (PE150) mode. Raw sequencing data were processed to remove low‐quality reads and adapter sequences; clean reads were aligned to the reference genome for downstream analyses, including differential gene expression, Gene Ontology (GO), Kyoto Encyclopedia of Genes and Genomes (KEGG) pathway enrichment, and gene set enrichment analysis (GSEA). Differentially expressed genes (DEGs) between groups were identified using DESeq2, with |log_2_ fold change| ≥ 1 and adjusted *p* < 0.01 considered significant. Volcano plots and heatmaps were generated to visualize DEG distributions.

siRNA‐mediated ATP7A knockdown and functional assays: Small interfering RNA (siRNA) specifically targeting ATP7A was designed and transfected into BMSCs using Lipofectamine 3000 (Thermo Fisher Scientific, USA) according to the manufacturer's instructions. After transfection, cells were cultured with β‐TCP@CQ scaffolds under HG conditions to evaluate the regulatory role of β‐TCP@CQ in the absence of ATP7A. Protein expression levels of ATP7A, superoxide dismutase 3 (SOD3), vascular endothelial growth factor receptor 1 (FLT1), RUNX2, phosphorylated phosphoinositide 3‐kinase (p‐PI3K), and phosphorylated protein kinase B (p‐Akt) were determined by WB analysis as described above. In parallel, ALP staining was performed to assess osteogenic activity across treatment groups.

### 5.11 In Vivo Therapeutic Efficacy of CQ Coatings in Diabetic Bone Regeneration

Cranial defect model and scaffold implantation in type 2 diabetic rats: All animal experiments were approved by the Ethics Committee of the Wenzhou Institute, University of Chinese Academy of Sciences (WIUCAS; approval no. WIUCAS24101001). 6‐week‐old male Wistar rats were used to establish a type 2 diabetes mellitus (T2DM) model by feeding a high‐fat diet followed by intraperitoneal injection of streptozotocin (STZ). Successful establishment of the diabetic phenotype was confirmed by intraperitoneal glucose tolerance test (IPGTT) and intraperitoneal insulin tolerance test (IPITT). Briefly, for the IPGTT, rats were fasted for 12 h and intraperitoneally injected with 50% (w/v) glucose (2.0 g/kg). Blood glucose levels were measured every 30 min for 2 h. For the IPITT, rats were fasted for 4 h, intraperitoneally injected with insulin (0.75 U/kg, 0.2 mL), and blood glucose levels were monitored every 30 min for 2 h. After confirmation of the diabetic phenotype, standard cranial defect surgery was performed. Rats were anesthetized with isoflurane and placed in the supine position. A midline sagittal incision was made, and the periosteum was bluntly dissected to expose the calvaria. A circular, full‐thickness critical‐sized defect (5 mm in diameter) was created using a trephine bur. Animals were randomly assigned to three groups: blank control (defect only), β‐TCP scaffold implantation, and β‐TCP@CQ scaffold implantation.

Micro‐CT analysis: At 8 weeks postimplantation, calvarial samples were harvested and fixed in 4% paraformaldehyde. micro‐CT (Skyscan 1276; Bruker, Belgium) was performed at 70 kV and 112 µA, with a matrix size of 1024 and a slice thickness of 0.048 mm. 3D reconstructions were generated using CTAn software, with scaffolds and new bone shown in different colors. The region of interest (ROI) was defined as the original defect (5 mm diameter), and bone mineral density (BMD), bone volume fraction (BV/TV), and trabecular thickness (Tb.Th) were quantified.

Histological analysis: Samples were fixed in 4% paraformaldehyde for 48 h, followed by decalcification in 10% ethylenediaminetetraacetic acid (EDTA, pH 7.4), with solution changes every 72 h for 4–6 weeks until complete decalcification. Decalcified tissues were dehydrated through graded ethanol, cleared in xylene, and embedded in paraffin. Sections of 5 µm thickness were cut along the sagittal or coronal plane.

For hematoxylin and eosin (H&E) staining, sections were deparaffinized, stained with hematoxylin for 5 min, rinsed and blued, and counterstained with eosin for 2 min. After dehydration and clearing, sections were mounted with neutral resin. Stained sections were examined under a light microscope to assess inflammatory cell infiltration, new bone formation, and tissue integrity.

For Masson's trichrome staining, sections were stained with Weigert's iron hematoxylin to visualize nuclei, differentiated in acidic ethanol, stained with Biebrich scarlet‐acid fuchsin for 3 min, and counterstained with aniline blue for 5 min to visualize collagen fibers. After brief differentiation in 1% acetic acid, followed by dehydration and clearing, sections were mounted with neutral resin. The proportion of collagen‐positive area was quantified using ImageJ software to evaluate collagen deposition in the bone matrix.

For immunohistochemical staining, antigen retrieval was performed in citrate buffer (pH 6.0) by microwave heating for 15 min, followed by cooling to room temperature. Endogenous peroxidase activity was blocked with 3% hydrogen peroxide for 10 min, and nonspecific binding was blocked with 5% goat serum for 1 h at room temperature. Sections were incubated overnight at 4°C with primary antibodies against OCN, RUNX2, CD31, FLT1, ATP7A, SOD3, p‐PI3K, and p‐Akt. After washing, sections were incubated with HRP‐conjugated secondary antibodies for 1 h at 37°C. Visualization was performed with DAB substrate for 3–5 min, counterstained with hematoxylin, dehydrated, and mounted. Positive signals appeared brown and were imaged under a light microscope. Quantitative analysis of the target regions was performed using ImageJ software.

### 5.12 Statistical Analysis

Data are expressed as mean ± standard error of the mean (SEM). For comparisons across multiple groups, statistical analysis was performed using one‐way ANOVA followed by Tukey's multiple comparisons test. A *p*‐value below 0.05 was considered statistically significant, with significance levels indicated as follows: *p < 0.05, **p < 0.01, and ***p < 0.001. All statistical computations were carried out using GraphPad Prism 9.0 software.

## Conflicts of Interest

The authors declare no conflicts of interest.

## Author Contributions

C.Y., C.D., L.S., and Z.L. contributed equally to this work. C.Y., C.D., L.F., and Z.L. conducted the study; L.H. and Q.J. assisted in material preparation; C.Z. and Y.W. assisted in data analysis; H.C. and J.C. guided the mechanistic investigation; C.Y. and Z.Z. analyzed the data, prepared figures, and wrote the manuscript; C.Y. and J.Y. designed and supervised the overall study.

## Supporting information




**Supporting File**: advs73493‐sup‐0001‐SuppMat.docx.

## Data Availability

The data that support the findings of this study are available from the corresponding author upon reasonable request.

## References

[1] C. Herder , C. Pritlove , N. Chaturvedi , and H. Mulder , “Diabetes Prevention and Treatment: A Global Perspective,” Diabetologia 68 (2025): 2303–2307. 40796982 10.1007/s00125-025-06511-6 PMC12534300

[2] S. Marino and T. Bellido , “PTH Receptor Signalling, Osteocytes and Bone Disease Induced by Diabetes Mellitus,” Nature Reviews Endocrinology 20 (2024): 661. 10.1038/s41574-024-01014-7 PMC13001153 39020007

[3] A. Lopez‐de‐Andrés , R. Jiménez‐García , I. Jiménez‐Trujillo , et al., “Incidence, Surgical Procedures, and Outcomes of Hip Fracture Among Elderly Type 2 Diabetic and Non‐Diabetic Patients in Spain (2004–2013),” Osteoporosis International 27 (2016): 605. 26318760 10.1007/s00198-015-3305-9

[4] A. V. Schwartz , “Epidemiology of Fractures in Type 2 Diabetes,” Bone 82 (2016): 2. 26027505 10.1016/j.bone.2015.05.032

[5] B. Wildemann , A. Ignatius , F. Leung , et al., “Non‐Union Bone Fractures,” Nature Reviews Disease Primers 7 (2021): 57. 10.1038/s41572-021-00289-8 34354083

[6] W. Zhou , Y. Liu , X. Nie , et al., “Peptide‐ Based Inflammation‐Responsive Implant Coating Sequentially Regulates Bone Regeneration to Enhance Interfacial Osseointegration,” Nature Communications 16 (2025): 3283. 10.1038/s41467-025-58444-8 PMC11973180 40189598

[7] M. Li , Y. Fan , M. Ran , et al., “Hydrogel Coatings of Implants for Pathological Bone Repair,” Advanced Healthcare Materials 13 (2024): 2401296. 10.1002/adhm.202401296 38794971

[8] Y. Li , Y. Liu , S. Liu , et al., “Diabetic Vascular Diseases: Molecular Mechanisms and Therapeutic Strategies,” Signal Transduction and Targeted Therapy 8 (2023): 152. 37037849 10.1038/s41392-023-01400-z PMC10086073

[9] X. Li , E. Xie , S. Sun , et al., “Flavonoids for Gastrointestinal Tract Local and Associated Systemic Effects: A Review of Clinical Trials and Future Perspectives,” Journal of Advanced Research 77 (2025): 15–41. 39798849 10.1016/j.jare.2025.01.014 PMC12627392

[10] D. Bellavia , E. Dimarco , V. Costa , et al., “Flavonoids in Bone Erosive Diseases: Perspectives in Osteoporosis Treatment,” Trends in Endocrinology & Metabolism 32 (2021): 76. 33288387 10.1016/j.tem.2020.11.007

[11] E. Rodríguez‐Arce and M. Saldías , “Antioxidant Properties of Flavonoid Metal Complexes and Their Potential Inclusion in the Development of Novel Strategies for the Treatment Against Neurodegenerative Diseases,” Biomedicine & Pharmacotherapy 143 (2021): 112236. 34649360 10.1016/j.biopha.2021.112236

[12] Z. Zhang , D. Chang , Z. Zeng , et al., “CuCS/Cur Composite Wound Dressings Promote Neuralized Skin Regeneration by Rebuilding the Nerve Cell “Factory” in Deep Skin Burns,” Materials Today Bio 26 (2024): 101075. 10.1016/j.mtbio.2024.101075 PMC11087995 38736614

[13] Q. Zhao , Y. Ni , H. Wei , et al., “Ion Incorporation into Bone Grafting Materials,” Periodontology 2000 94 (2024): 213–230. 37823468 10.1111/prd.12533

[14] L. Wang , S. Jiang , J. Zhou , et al., “From Hard Tissues to Beyond: Progress and Challenges of Strontium‐Containing Biomaterials in Regenerative Medicine Applications,” Bioactive Materials 49 (2025): 85. 40124596 10.1016/j.bioactmat.2025.02.039 PMC11928986

[15] Y. Zhu , X. Zhang , G. Chang , S. Deng , and H. F. Chan , “Bioactive Glass in Tissue Regeneration: Unveiling Recent Advances in Regenerative Strategies and Applications,” Advanced Materials 37 (2025): 2312964. 39014919 10.1002/adma.202312964 PMC11733714

[16] J. S. Lee , J. S. Lee , M. S. Lee , et al., “Plant Flavonoid‐Mediated Multifunctional Surface Modification Chemistry: Catechin Coating for Enhanced Osteogenesis of Human Stem Cells,” Chemistry of Materials 29 (2017): 4375.

[17] J. Saiz‐Poseu , J. Mancebo‐Aracil , F. Nador , F. Busqué , and D. Ruiz‐Molina , “The Chemistry Behind Catechol‐Based Adhesion,” Angewandte Chemie International Edition 58 (2019): 696. 29573319 10.1002/anie.201801063

[18] P. K. Walencik , R. Choińska , E. Gołębiewska , and M. Kalinowska , “Metal–Flavonoid Interactions—From Simple Complexes to Advanced Systems,” Molecules (Basel, Switzerland) 29 (2024): 2573. 38893449 10.3390/molecules29112573 PMC11173564

[19] H. Ejima , J. J. Richardson , K. Liang , et al., “One‐Step Assembly of Coordination Complexes for Versatile Film and Particle Engineering,” Science 341 (2013): 154. 23846899 10.1126/science.1237265

[20] S. Yang , Y. Zhu , C. Ji , et al., “A Five‐in‐One Novel MOF‐Modified Injectable Hydrogel With Thermo‐Sensitive and Adhesive Properties for Promoting Alveolar Bone Repair in Periodontitis: Antibacterial, Hemostasis, Immune Reprogramming, Pro‐osteo‐/Angiogenesis and Recruitment,” Bioactive Materials 41 (2024): 239. 39149594 10.1016/j.bioactmat.2024.07.016 PMC11324614

[21] X. Yu , M. Gholipourmalekabadi , X. Wang , C. Yuan , and K. Lin , “Three‐ Dimensional Bioprinting Biphasic Multicellular Living Scaffold Facilitates Osteochondral Defect Regeneration,” Interdisciplinary Materials 3 (2024): 738.

[22] L. Bai , P. Song , and J. Su , “Bioactive Elements Manipulate Bone Regeneration,” Biomaterials Translational 4 (2023): 248–269. 38282709 10.12336/biomatertransl.2023.04.005 PMC10817798

[23] K. Jomova , S. Y. Alomar , R. Valko , et al., “Flavonoids and Their Role in Oxidative Stress, Inflammation, and Human Diseases,” Chemico‐Biological Interactions 413 (2025): 111489. 40147618 10.1016/j.cbi.2025.111489

[24] K. Xu , W. Fei , W. Gao , et al., “SOD3 Regulates FLT1 to Affect Bone Metabolism by Promoting Osteogenesis and Inhibiting Adipogenesis Through PI3K/AKT and MAPK Pathways,” Free Radical Biology and Medicine 212 (2024): 65. 38141889 10.1016/j.freeradbiomed.2023.12.021

[25] Y. Wang , H. Zhang , Y. Hu , Y. Jing , Z. Geng , and J. Su , “Bone Repair Biomaterials: A Perspective From Immunomodulation,” Advanced Functional Materials 32 (2022): 2208639.

[26] R. Wang , S. Su , Z. Chen , and F. Zhou , “Type 2 Diabetes Mellitus Mediated Oxidative Stress in Bone Tissues and Novel Challenges for Biomaterials,” Advanced Therapeutics 7 (2024): 2300231.

[27] J. I. Kourie , “Interaction of Reactive Oxygen Species with Ion Transport Mechanisms,” American Journal of Physiology‐Cell Physiology 275 (1998): C1. 10.1152/ajpcell.1998.275.1.C1 9688830

[28] D. Jia , L. Liu , W. Liu , J. Li , X. Jiang , and Y. Xin , “Copper Metabolism and Its Role in Diabetic Complications: A Review,” Pharmacological Research 206 (2024): 107264. 38876443 10.1016/j.phrs.2024.107264

[29] C. Liu , B. Yu , Z. Zhang , et al., “LIPUS Activated Piezoelectric pPLLA/SrSiO3 Composite Scaffold Promotes Osteochondral Regeneration Through P2RX1 Mediated Ca2+ Signaling Pathway,” Biomaterials 317 (2025): 123084. 39754966 10.1016/j.biomaterials.2025.123084

[30] L. Deng , L. Huang , H. Pan , et al., “3D Printed Strontium–Zinc‐Phosphate Bioceramic Scaffolds With Multiple Biological Functions for Bone Tissue Regeneration,” Journal of Materials Chemistry B 11 (2023): 5469. 36723376 10.1039/d2tb02614g

[31] L. Huang , Y. Jiao , H. Xia , et al., “Strontium Zinc Silicate Simultaneously Alleviates Osteoporosis and Sarcopenia in Tail‐Suspended Rats via Piezo1‐Mediated Ca2+ Signaling,” Journal of Orthopaedic Translation 48 (2024): 146. 39229332 10.1016/j.jot.2024.07.014 PMC11369381

[32] Y. Zhou , C. Wu , and J. Chang , “Bioceramics to Regulate Stem Cells and Their Microenvironment for Tissue Regeneration,” Materials Today 24 (2019): 41.

[33] A. Arteaga , J. Qu , S. Haynes , B. G. Webb , J. LaFontaine , and D. C. Rodrigues , “Diabetes as a Risk Factor for Orthopedic Implant Surface Performance: A Retrieval and In Vitro Study,” Journal of Bio‐ and Tribo‐Corrosion 7 (2021): 51. 34150468 10.1007/s40735-021-00486-8 PMC8211117

[34] S. Selvaraj , S. Krishnaswamy , V. Devashya , S. Sethuraman , and U. M. Krishnan , “Flavonoid–Metal Ion Complexes: A Novel Class of Therapeutic Agents,” Medicinal Research Reviews 34 (2014): 677. 24037904 10.1002/med.21301

[35] C. Yang , H. Ma , Z. Wang , et al., “3D Printed Wesselsite Nanosheets Functionalized Scaffold Facilitates NIR‐II Photothermal Therapy and Vascularized Bone Regeneration,” Advanced Science 8 (2021): 2100894. 34396718 10.1002/advs.202100894 PMC8529444

[36] P. Barć , M. Antkiewicz , K. Frączkowska‐Sioma , et al., “Two‐Stage Gene Therapy (VEGF, HGF and ANG1 Plasmids) as Adjunctive Therapy in the Treatment of Critical Lower Limb Ischemia in Diabetic Foot Syndrome,” International Journal of Environmental Research and Public Health 19 (2022): 12818. 36232122 10.3390/ijerph191912818 PMC9564889

[37] Y. Chen , Y. Zhou , J. Lin , and S. Zhang , “Challenges to Improve Bone Healing Under Diabetic Conditions,” Frontiers in Endocrinology 13 (2022): 861878. 35418946 10.3389/fendo.2022.861878 PMC8996179

[38] M. R. Islam , S. Akash , M. H. Jony , et al., “Exploring the Potential Function of Trace Elements in Human Health: A Therapeutic Perspective,” Molecular and Cellular Biochemistry 478 (2023): 2141. 36637616 10.1007/s11010-022-04638-3

[39] Y. Wang , D. Li , K. Xu , G. Wang , and F. Zhang , “Copper Homeostasis and Neurodegenerative Diseases,” Neural Regeneration Research 20 (2025): 3124. 39589160 10.4103/NRR.NRR-D-24-00642 PMC11881714

[40] S. Anwar , T. Sarwar , A. A. Khan , and A. H. Rahmani , “Therapeutic Applications and Mechanisms of Superoxide Dismutase (SOD) in Different Pathogenesis,” Biomolecules 15 (2025): 1130. 40867576 10.3390/biom15081130 PMC12384489

[41] L. Lu , Y. Wu , C. Lu , et al., “Molecular Mechanism of Flt‐1 Protein and the Regulation of Monocytes Modulate Endothelial Cell in Wound Healing Sites via PGF/FLT1 Signaling,” International Journal of Biological Macromolecules 307 (2025): 142192. 40101823 10.1016/j.ijbiomac.2025.142192

[42] Z. Meng , J. Liu , Z. Feng , et al., “N‐Acetylcysteine Regulates Dental Follicle Stem Cell Osteogenesis and Alveolar Bone Repair via ROS Scavenging,” Stem Cell Research & Therapy 13 (2022): 466. 36076278 10.1186/s13287-022-03161-y PMC9461171

[43] G. Calabrese , A. Ardizzone , M. Campolo , S. Conoci , E. Esposito , and I. Paterniti , “Beneficial Effect of Tempol, a Membrane‐Permeable Radical Scavenger, on Inflammation and Osteoarthritis in In Vitro Models,” Biomolecules 11 (2021): 352. 33669093 10.3390/biom11030352 PMC7996488

[44] J. Yang , H. Pan , K. Sekimata , et al., “A New BMP Type 1 Receptor Kinase Inhibitor for Safe and Efficient Oral Treatment to Prevent Genetically Induced Heterotopic Ossification in Mice,” Bone 199 (2025): 117565. 40516669 10.1016/j.bone.2025.117565 PMC12332771

[45] D. Ash , V. Sudhahar , S.‐W. Youn , et al., “The P‐type ATPase Transporter ATP7A Promotes Angiogenesis by Limiting Autophagic Degradation of VEGFR2,” Nature Communications 12 (2021): 3091. 10.1038/s41467-021-23408-1 PMC8149886 34035268

[46] L. Liu , W. Liu , X. Dong , and Y. Sun , “A Multifunctional Theranostic Agent Based on Rhodamine‐Modified Copper‐Gallic Acid Nanoparticles Targeting Alzheimer's β‑amyloid Species,” Chemical Engineering Journal 513 (2025): 163063.

[47] Y. Xu , Y. Luo , Z. Weng , et al., “Microenvironment‐Responsive Metal‐Phenolic Nanozyme Release Platform With Antibacterial, ROS Scavenging, and Osteogenesis for Periodontitis,” ACS Nano 17 (2023): 18732. 37768714 10.1021/acsnano.3c01940

[48] X. Hu , J. He , L. Qiao , et al., “Multifunctional Dual Network Hydrogel Loaded With Novel Tea Polyphenol Magnesium Nanoparticles Accelerates Wound Repair of MRSA Infected Diabetes,” Advanced Functional Materials 34 (2024): 2312140.

[49] M. A. Kahlson and S. J. Dixon , “Copper‐induced Cell Death,” Science 375 (2022): 1231. 35298241 10.1126/science.abo3959

