# Supplementary material for: A Universal Metal–Flavonoid Coating Strategy: Engineering Biomaterials for Diabetic Bone Regeneration
Source: Adv Sci (Weinh). 2026 Jan 8;13(10):e22509. doi: 10.1002/advs.202522509 (PMC12915126; doi:10.1002/advs.202522509)
Supplement: Supplementary file 1 — Supporting File: advs73493‐sup‐0001‐SuppMat.docx. [file ADVS-13-e22509-s001.docx]

**Supporting Information**

**A Universal Metal-Flavonoid Coating Strategy: Engineering Biomaterials for Diabetic Bone Regeneration**

*Chen Yang^*^,* *Chenle Dong,* *Lefeng Su,* *Zhiqiang Liu, Lingyi Hu,* *Qishu Jin,* *Hao Chen,* *Chunlong Zhang,* *Yihao Wu,* *Jiang Chang,* *Zhaowenbin Zhang^*^,* *Jiandong Yuan^*^*

C. Yang, Z. Liu, L. Hu, and J. Yuan

Department of Orthopaedics, Joint Centre of Translational Medicine, The First Affiliated Hospital of Wenzhou Medical University, Wenzhou, Zhejiang 325035, China.

Email: cryangchen@suda.edu.cn; yuanjiandong@wzhospital.cn.

C. Yang, C. Dong, L. Su, Q. Jin, C. Zhang, Y. Wu, and J. Chang

Zhejiang Engineering Research Center for Tissue Repair Materials, Wenzhou Institute, University of Chinese Academy of Sciences, Wenzhou, Zhejiang 325000, China

C. Yang

Orthopedic Institute, The First Affiliated Hospital, Suzhou Medical College, Soochow University, Suzhou, Jiangsu 215000, China

C. Dong,

Institute of Reproductive Health, Tongji Medical College, Huazhong University of Science and Technology, Wuhan, Hubei 430030, China.

H. Chen,

Medical College, Yangzhou University, Yangzhou, Jiangsu 225001, China

Z. Zhang

^f^ State Key Laboratory of Advanced Fiber Materials, College of Biological Science and Medical Engineering, Donghua University, Shanghai 201620, China

Email: zhangzhaowb@dhu.edu.cn.

*Corresponding authors.

E-mail addresses:

cryangchen@suda.edu.cn (Chen Yang),

zhangzhaowb@dhu.edu.cn (Zhaowenbin Zhang),

yuanjiandong@wzhospital.cn (Jiandong Yuan).


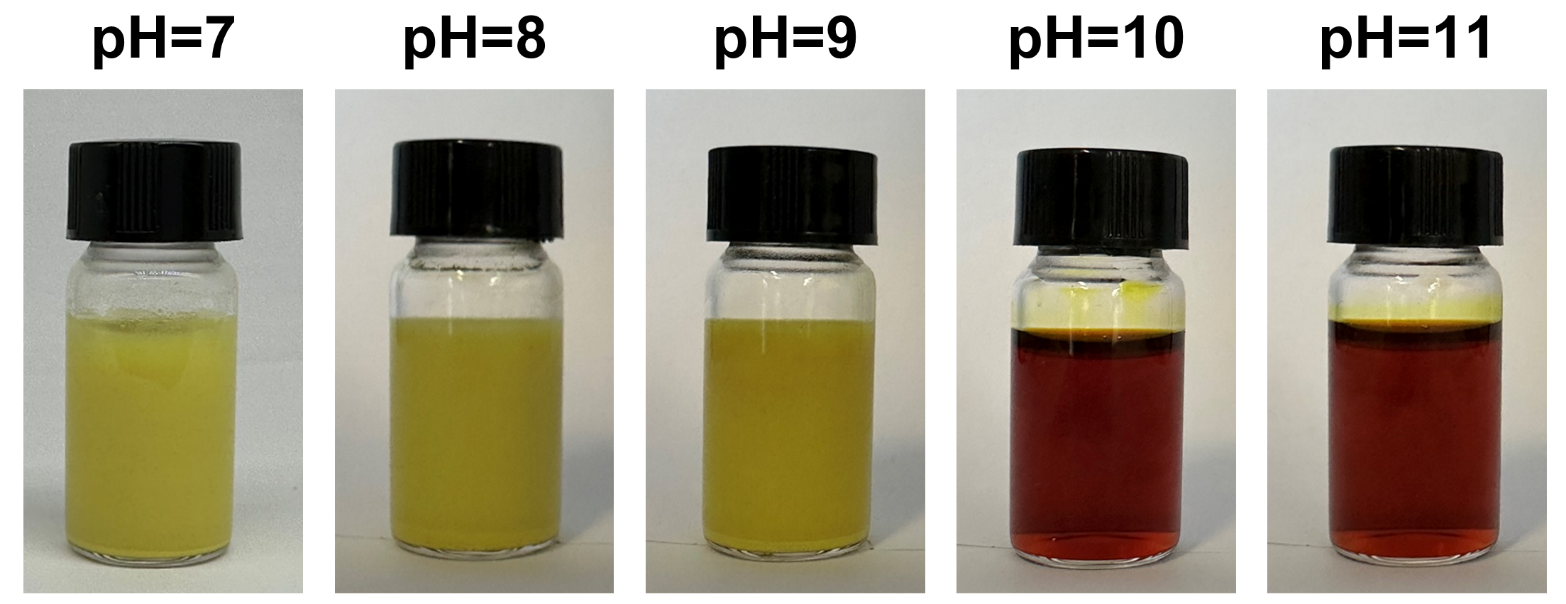


**Figure S1.** Solubility of quercetin in aqueous solution at different pH values.


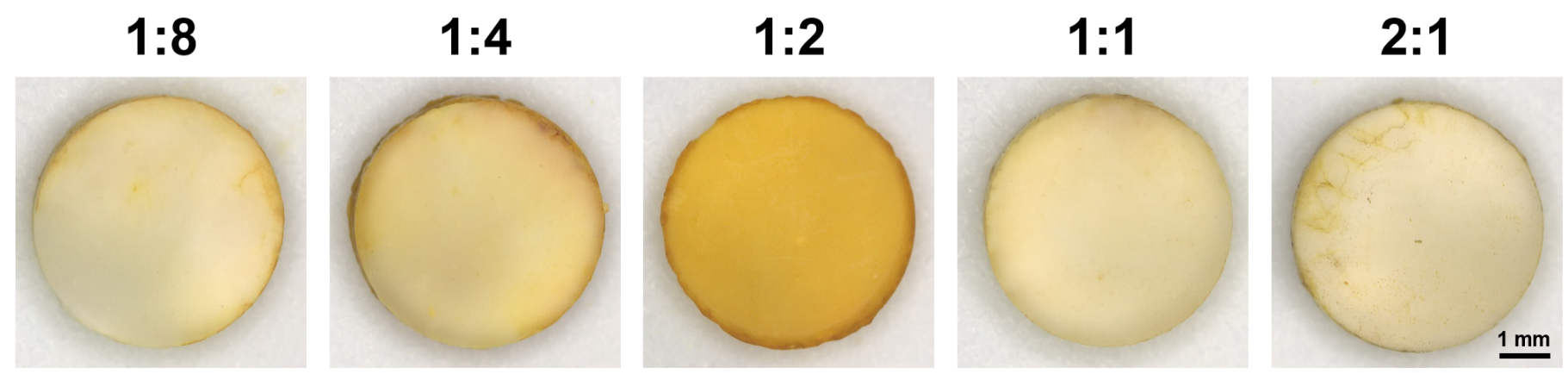


**Figure S2.** Formation of CQ coatings on the surface of β-TCP discs at different Cu-quercetin molar ratios.


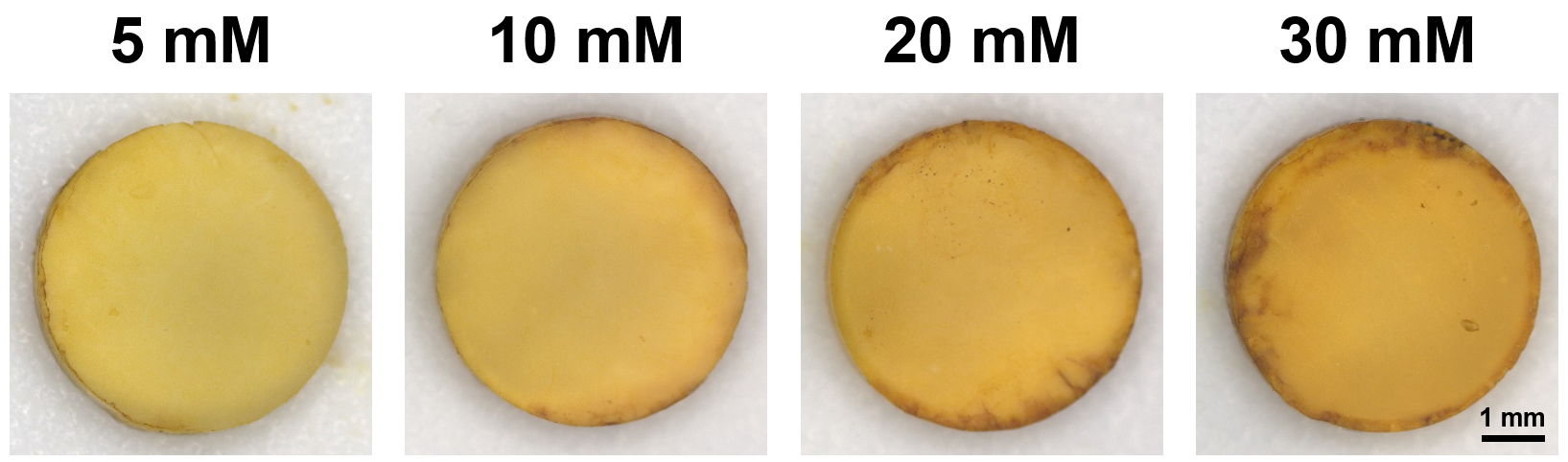


**Figure S3.** Formation of CQ coatings on the surface of β-TCP discs at different quercetin concentrations.


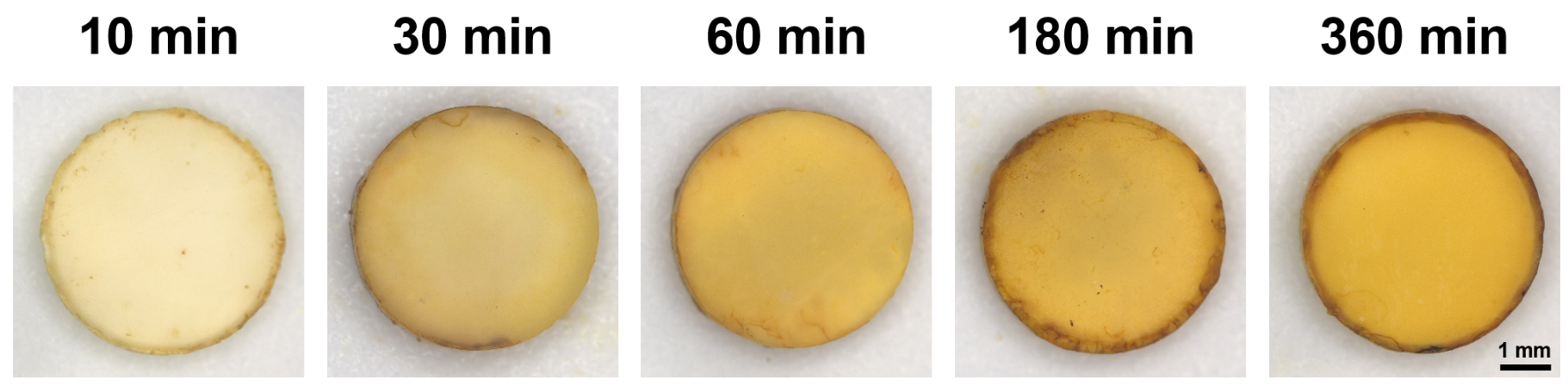


**Figure S4.** Formation of CQ coatings on the surface of β-TCP discs at different reaction times.


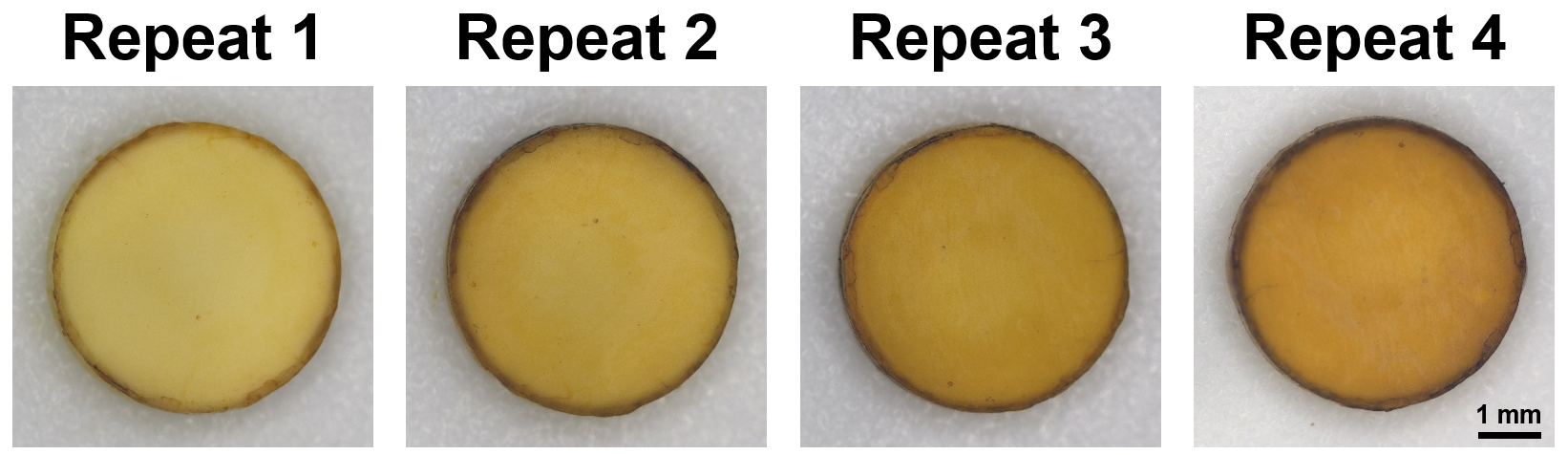


**Figure S5.** Formation of CQ coatings on the surface of β-TCP discs at different coating cycles.


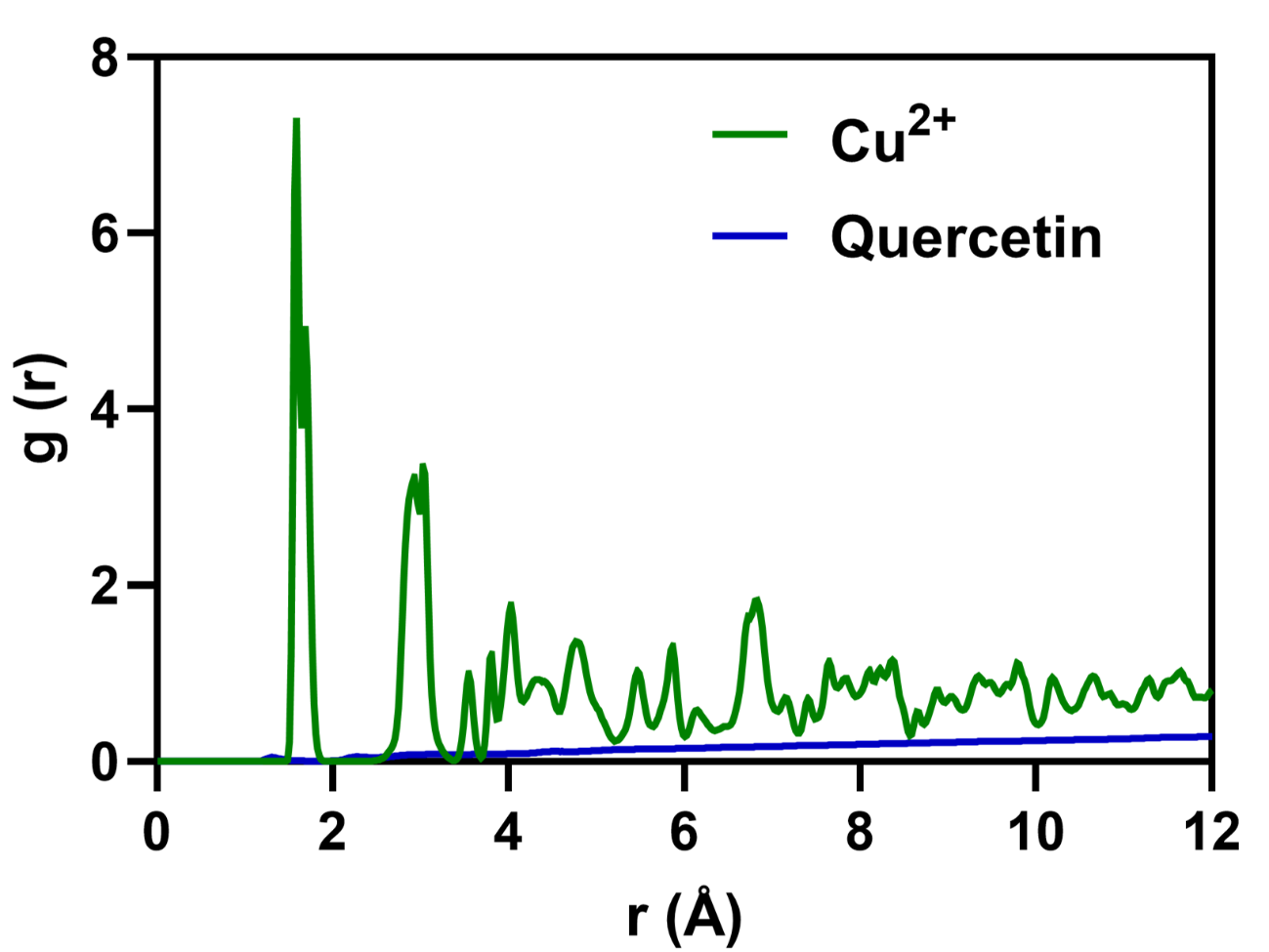


**Figure S6.** Molecular dynamics simulation of the radial distribution of Cu^2+^ and quercetin.


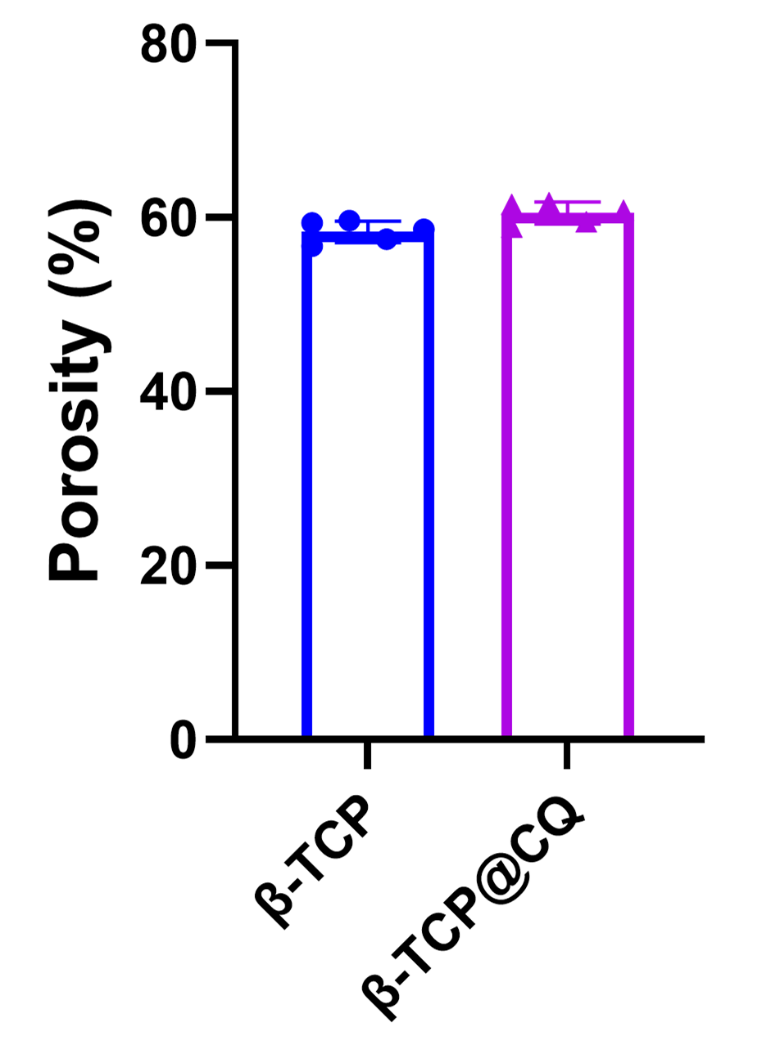


**Figure S7.** Porosity of β-TCP and β-TCP@CQ scaffolds. n=5.


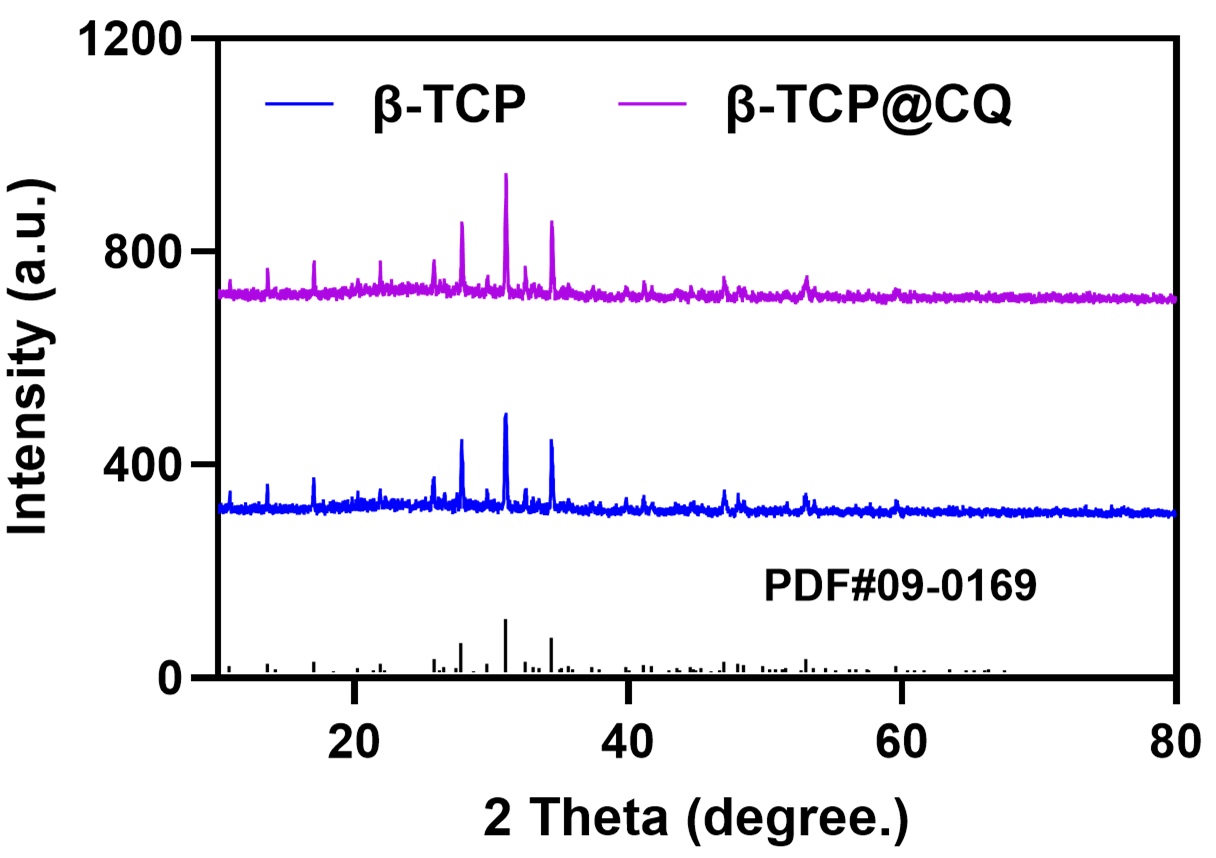


**Figure S8.** XRD spectra of β-TCP and β-TCP@CQ.


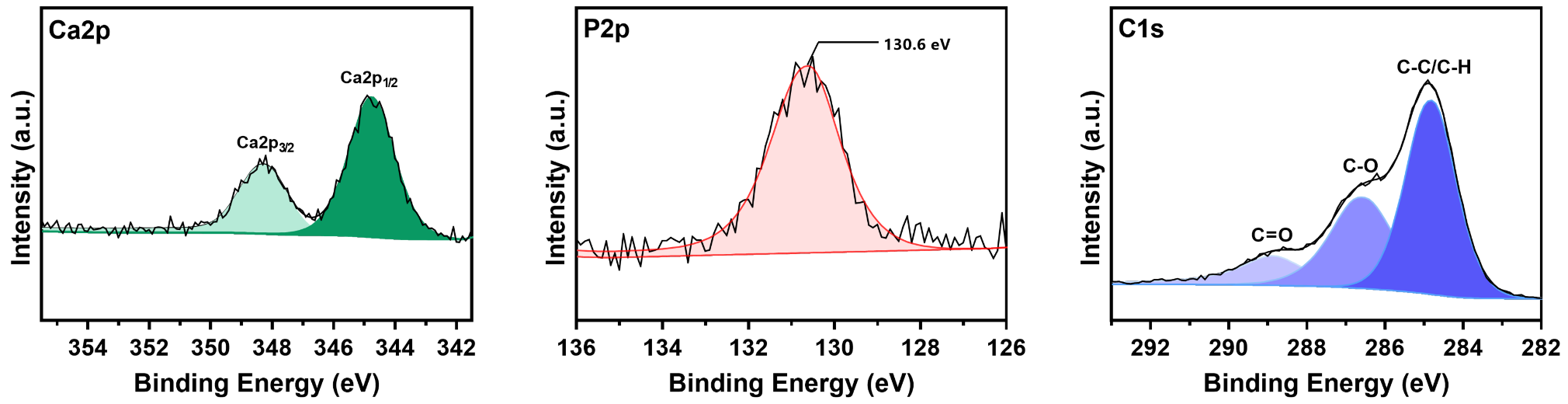


**Figure S9.** High resolution XPS spectrum of Ca 2p, P 2p and C 1s in β-TCP@CQ.


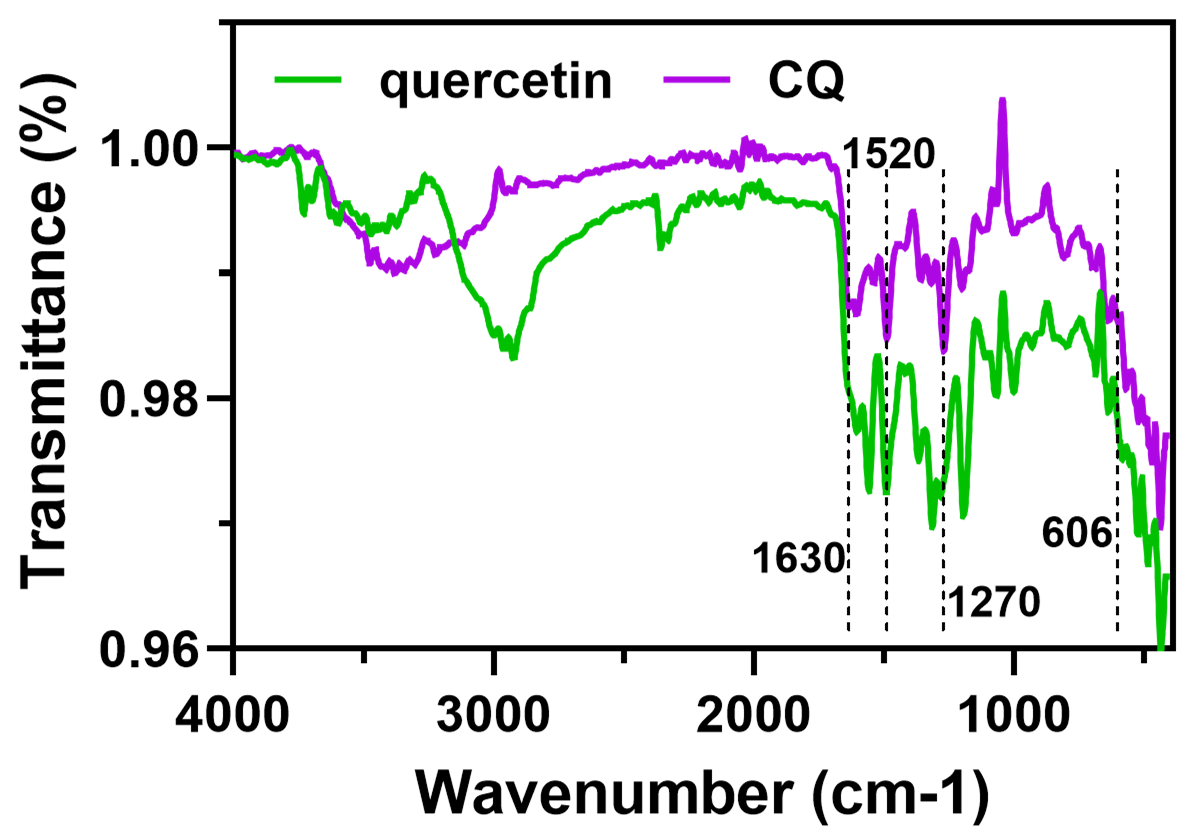


**Figure S10.** FTIR spectra of quercetin and CQ.


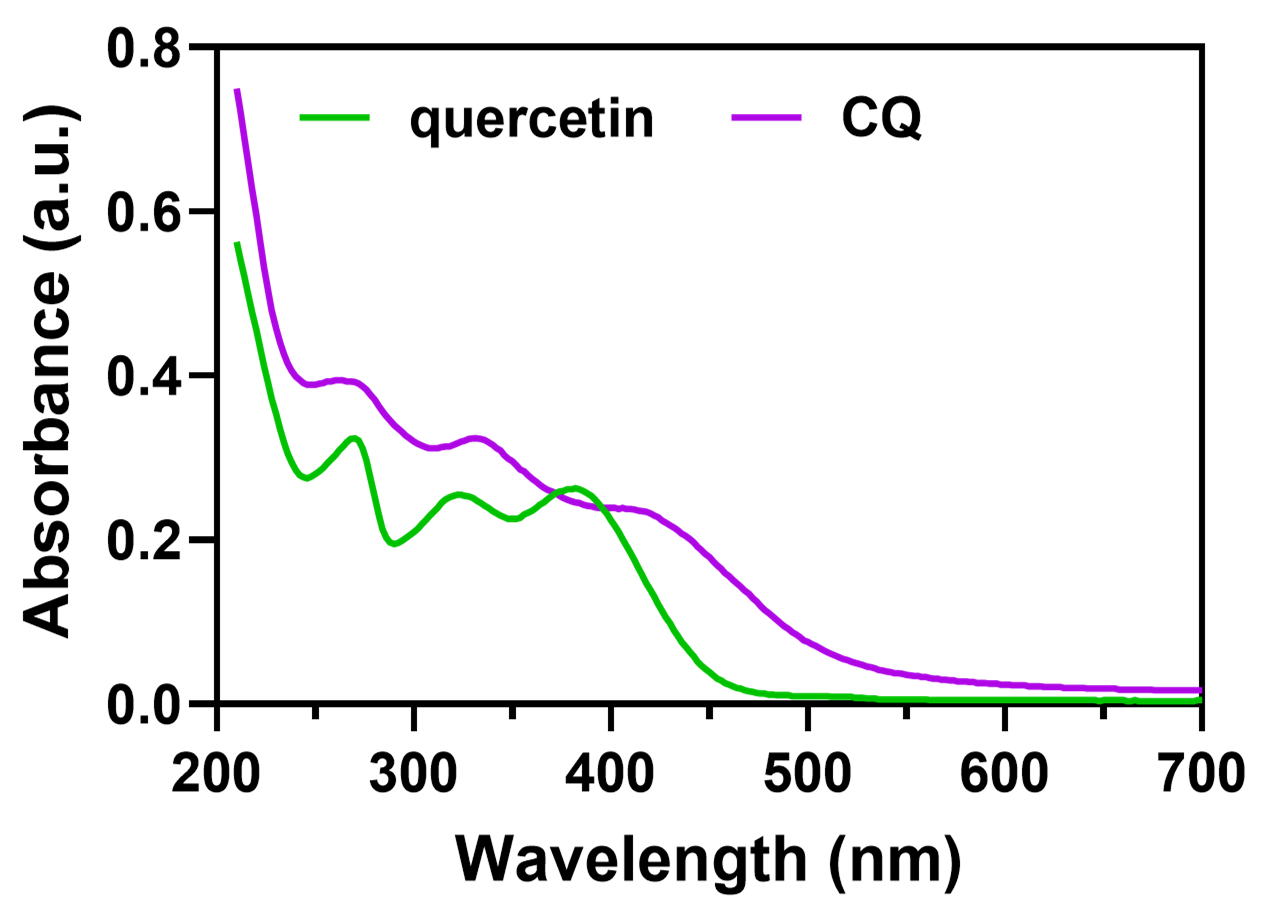


**Figure S11.** UV-Vis spectra of quercetin and CQ.


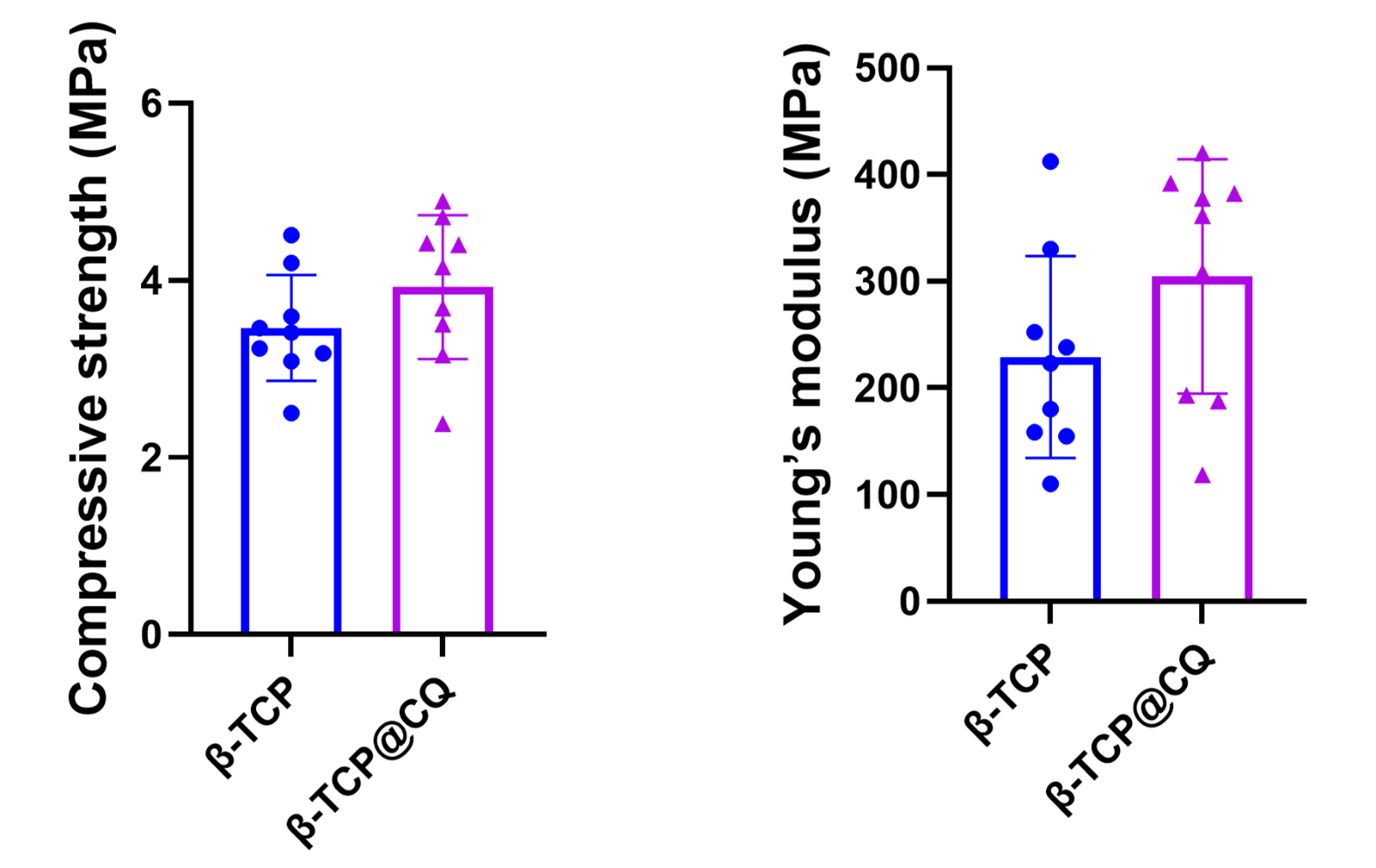


**Figure S12.** Compressive strength and Young’s modulus of β-TCP and β-TCP@CQ scaffolds. n=9.


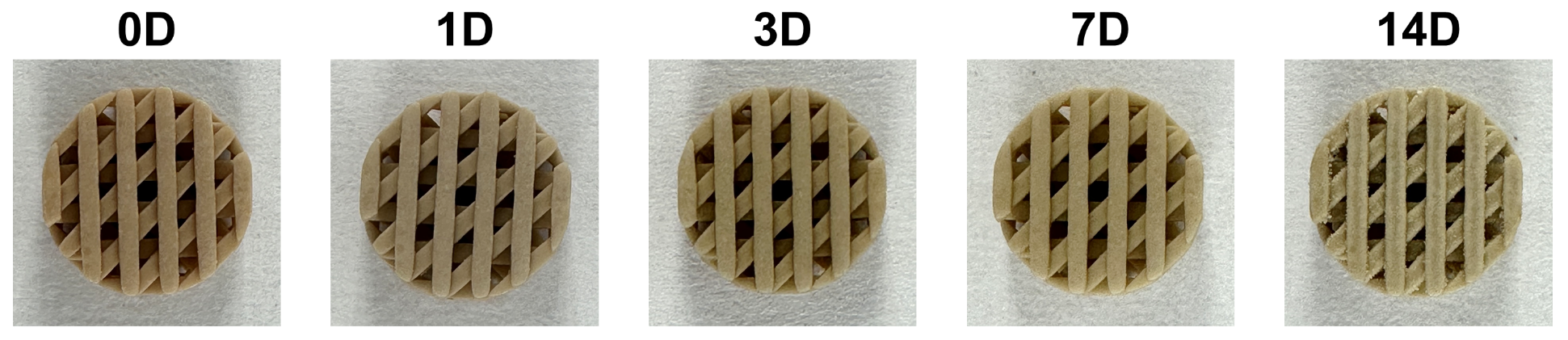


**Figure S13.** Macroscopic images of β-TCP@CQ scaffolds during the degradation process.


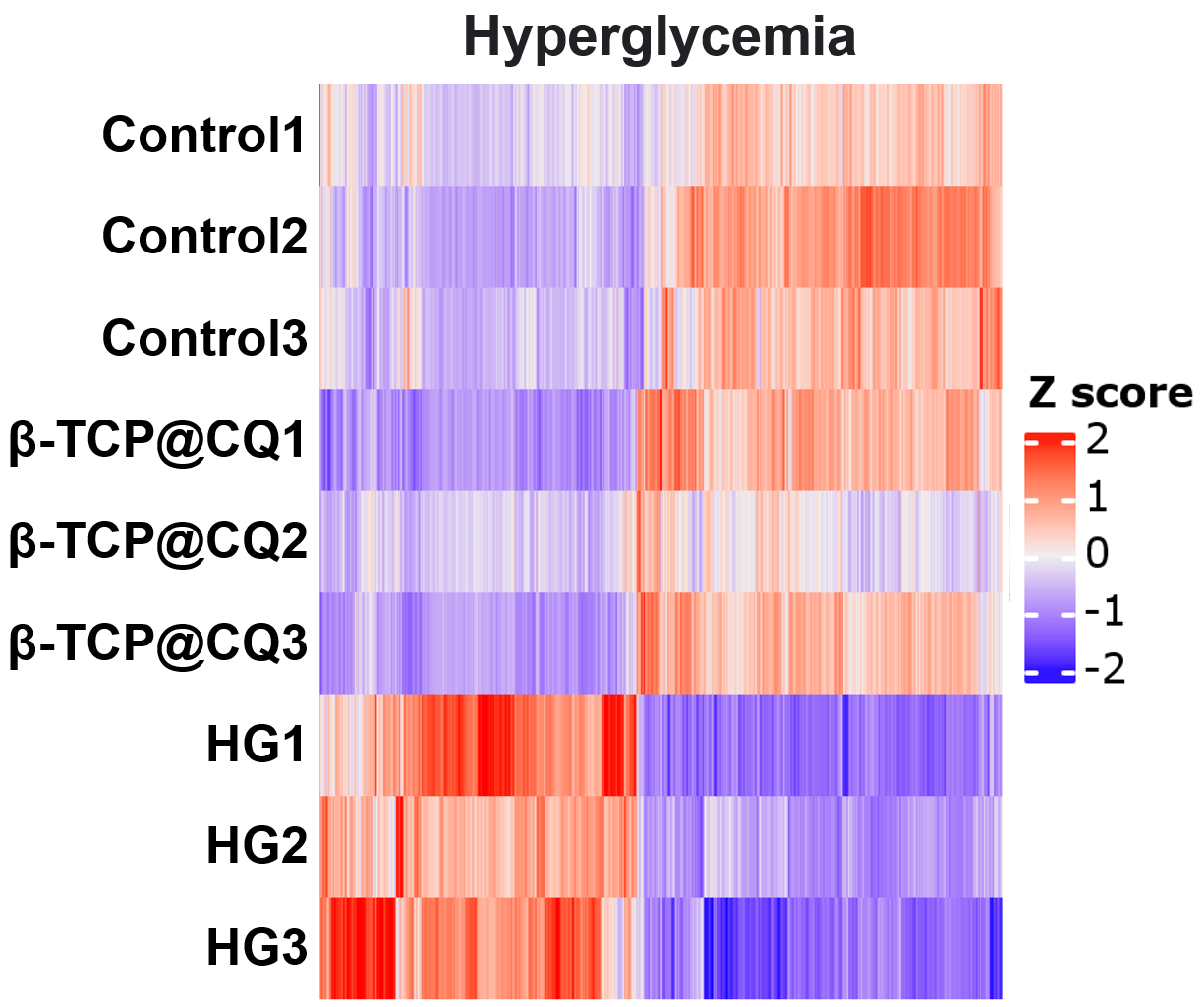


**Figure S14.** Heatmap of differential genes associated with hyperglycemia.


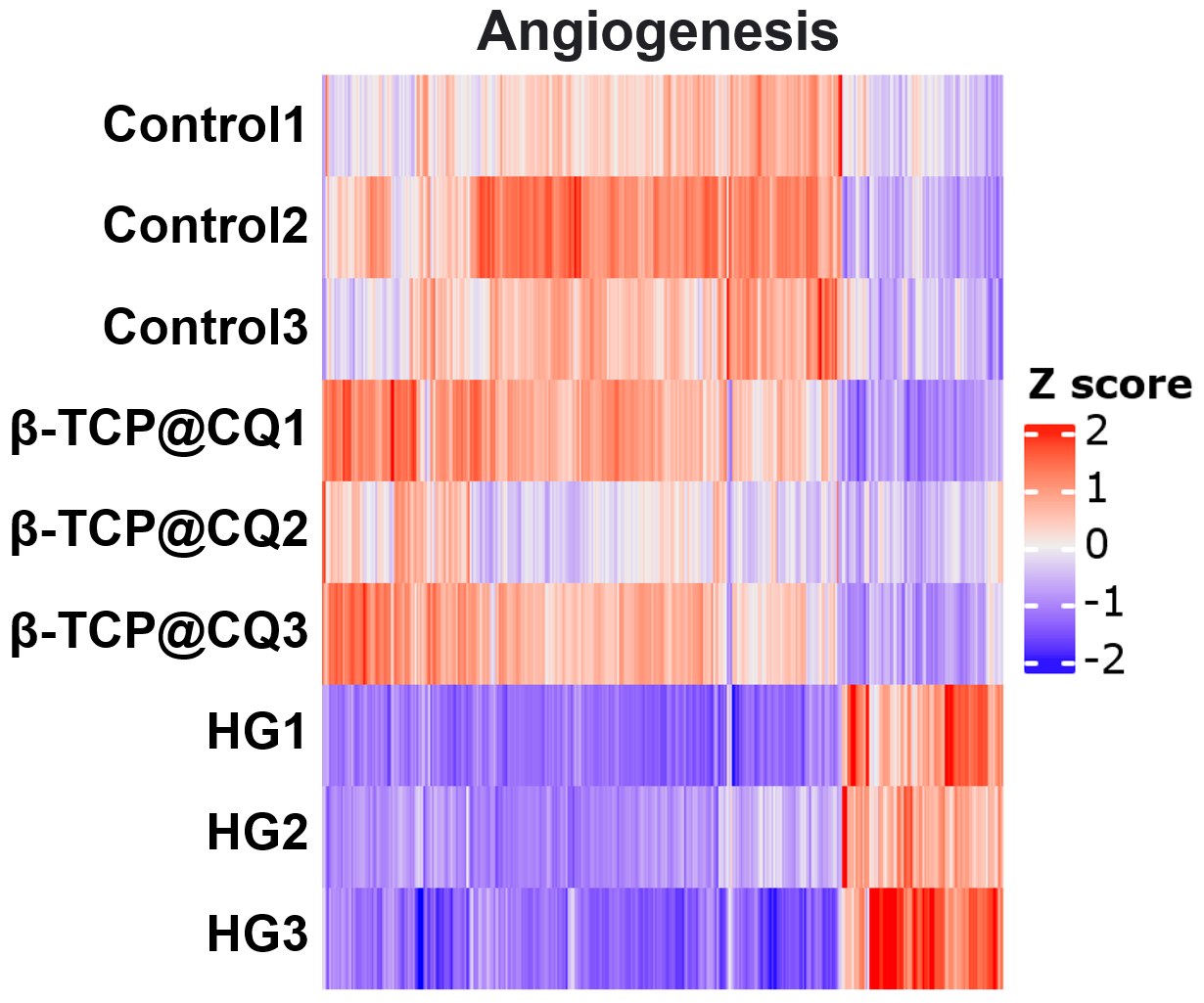


**Figure S15.** Heatmap of differential genes related to angiogenesis.


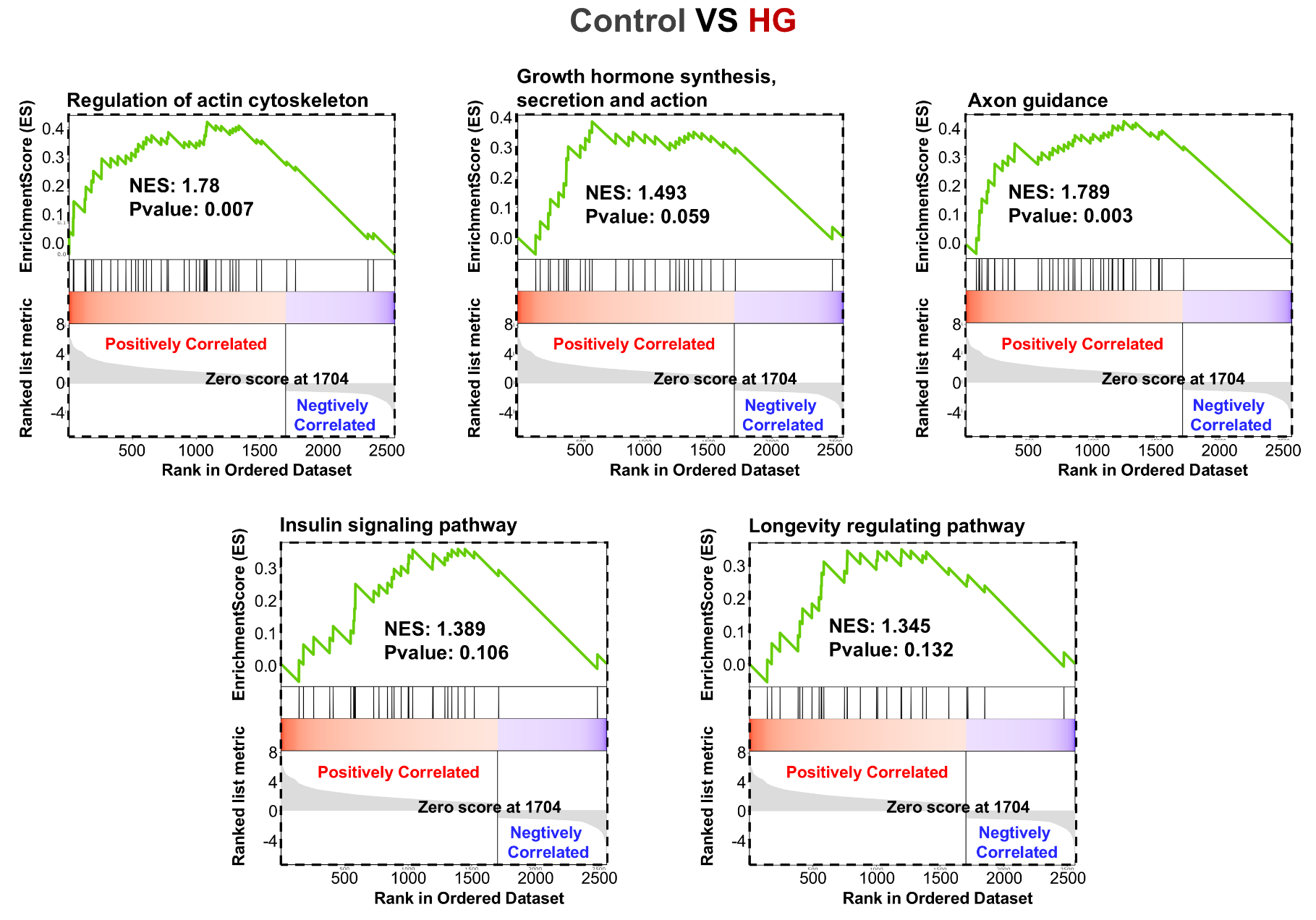


**Figure S16.** GSEA of the “Regulation of actin cytoskeleton”, “Growth hormone synthesis, secretion and action”, “Axon guidance”, “Insulin signaling pathway”, and “Longevity regulating pathway” pathways between the Control and HG groups.


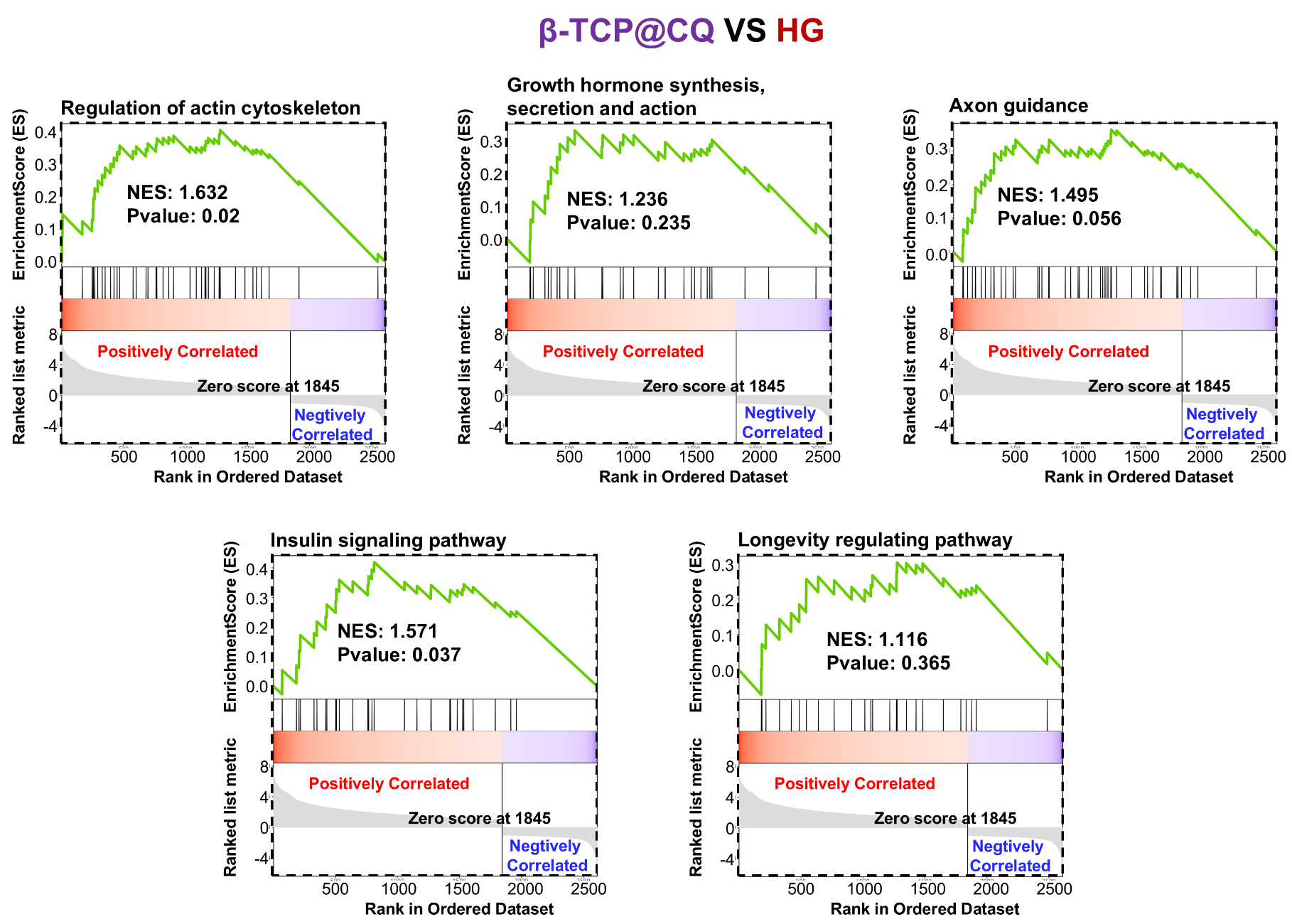


**Figure S17.** GSEA of the “Regulation of actin cytoskeleton”, “Growth hormone synthesis, secretion and action”, “Axon guidance”, “Insulin signaling pathway”, and “Longevity regulating pathway” pathways between the β-TCP@CQ group and HG group.


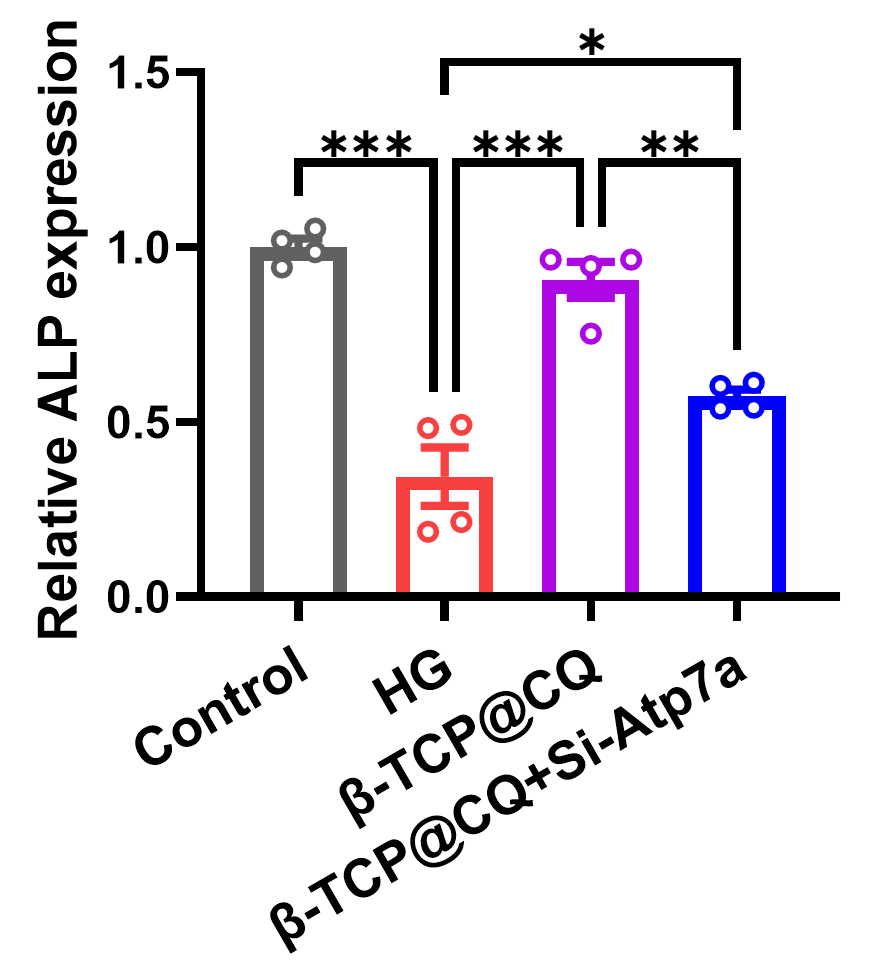


**Figure S18.** Relative ALP expression in HG-injured BMSCs treated with β-TCP@CQ in the presence of Atp7a knockdown mediated by siRNA. n=4.


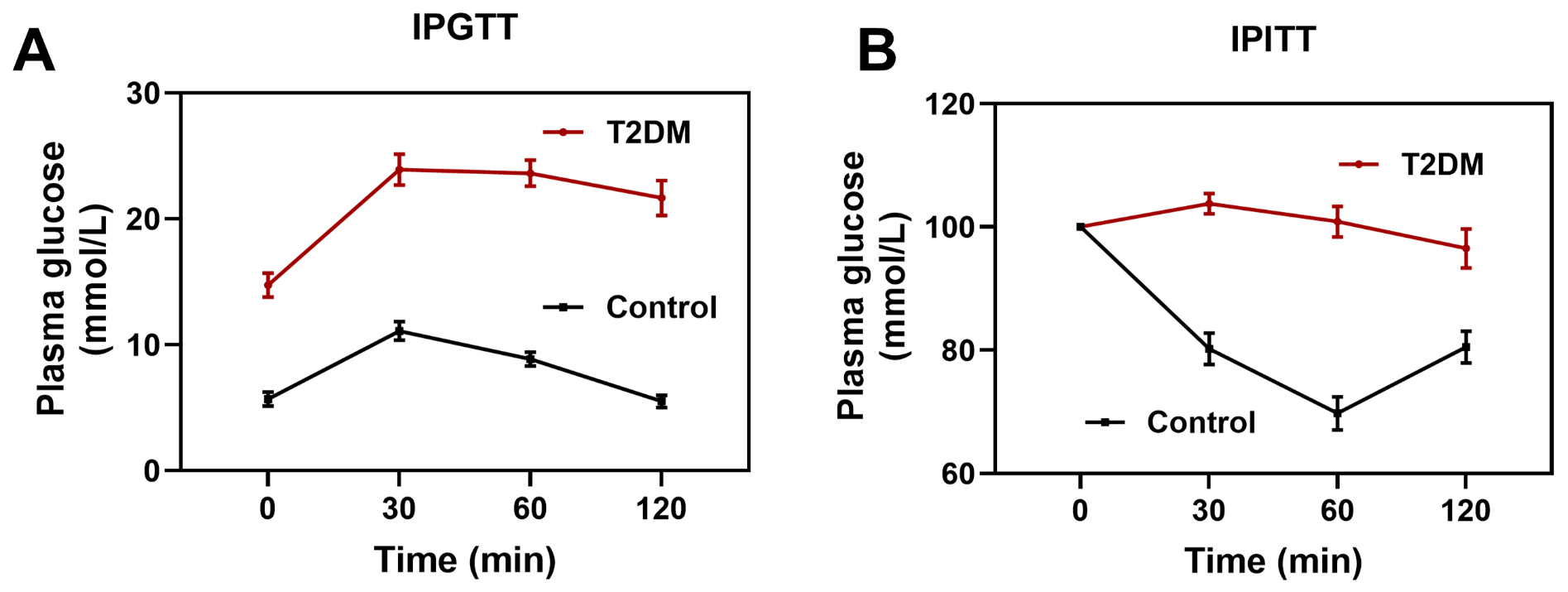


**Figure S19.** Evaluation of the establishment of a type 2 diabetes mellitus rat model through intraperitoneal glucose tolerance test (IPGTT) and intraperitoneal insulin tolerance test (IPITT). n=5.


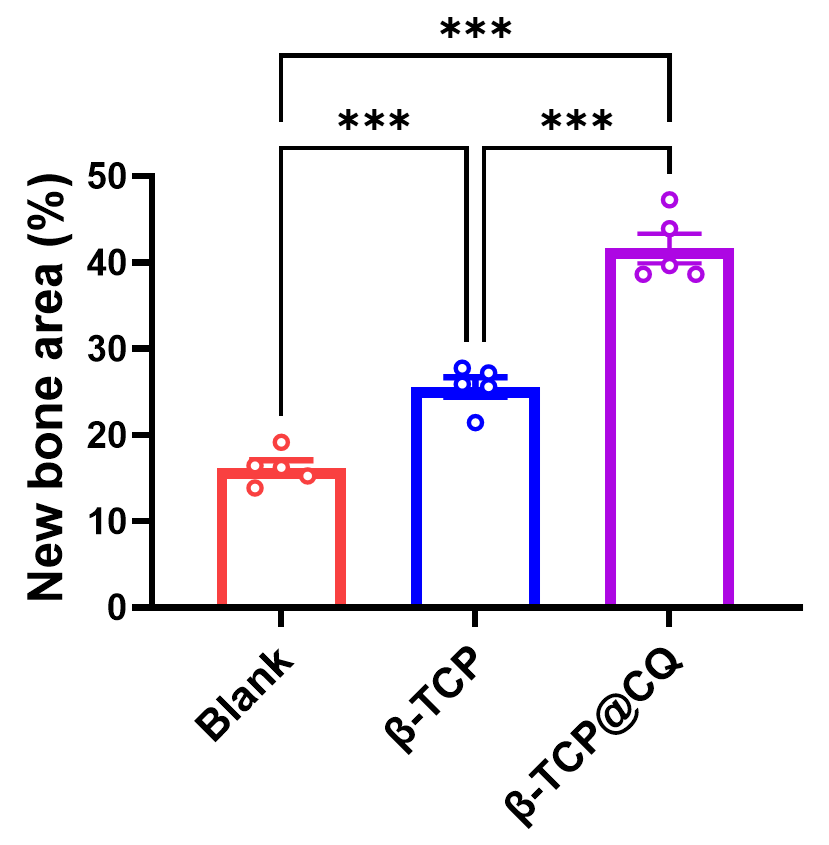


**Figure S20.** Quantitative analysis of new bone formation in the defect area following different treatments, assessed by H&E staining. n=5.


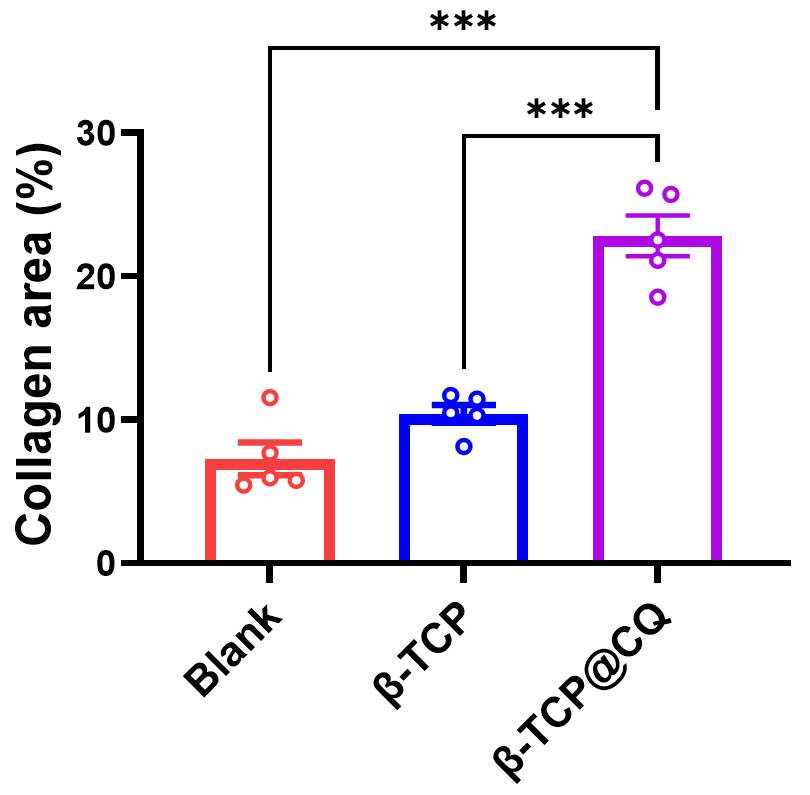


**Figure S21.** Quantitative analysis of collagen deposition in the defect area following different treatments, assessed by Masson trichrome staining. n=5.
